# *Chlamydia trachomatis* inhibits apoptosis in infected cells by targeting the pro-apoptotic proteins Bax and Bak

**DOI:** 10.1038/s41418-022-00995-0

**Published:** 2022-04-09

**Authors:** Collins Waguia Kontchou, Ian E. Gentle, Arnim Weber, Axel Schoeniger, Frank Edlich, Georg Häcker

**Affiliations:** 1 Institute of Medical Microbiology and Hygiene, Medical Center - University of Freiburg, Faculty of Medicine, 79104 Freiburg, Germany; 2 Veterinary Physiological Chemical Institute, Faculty of Veterinary Medicine, University of Leipzig, 04103 Leipzig, Germany; 3 BIOSS Centre for Biological Signalling Studies, University of Freiburg, 79104 Freiburg, Germany

**Keywords:** Protein quality control, Microbiology

## Abstract

Apoptosis acts in defense against microbial infection, and many infectious agents have developed strategies to inhibit host cell apoptosis. The human pathogen *Chlamydia trachomatis* (*Ctr*) is an obligate intracellular bacterium that strongly inhibits mitochondrial apoptosis of its human host cell but there is no agreement how the bacteria achieve this. We here provide a molecular analysis of chlamydial apoptosis-inhibition in infected human cells and demonstrate that the block of apoptosis occurs during the activation of the effectors of mitochondrial apoptosis, Bak and Bax. We use small-molecule Bcl-2-family inhibitors and gene targeting to show that previous models cannot explain the anti-apoptotic effect of chlamydial infection. Although the anti-apoptotic Bcl-2-family protein Mcl-1 was strongly upregulated upon infection, Mcl-1-deficient cells and cells where Mcl-1 was pharmacologically inactivated were still protected. *Ctr*-infection could inhibit both Bax- and Bak-induced apoptosis. Apoptotic Bax-oligomerization and association with the outer mitochondrial membrane was reduced upon chlamydial infection. Infection further inhibited apoptosis induced conformational changes of Bak, as evidenced by changes to protease sensitivity, oligomerization and release from the mitochondrial porin VDAC2. Mitochondria isolated from *Ctr*-infected cells were protected against the pro-apoptotic Bcl-2-family proteins Bim and tBid but this protection was lost upon protease digestion. However, the protective effect of *Ctr*-infection was reduced in cells lacking the Bax/Bak-regulator VDAC2. We further found that OmpA, a porin of the outer membrane of *Ctr*, associated upon experimental expression with mitochondria and inhibited apoptosis, phenocopying the effect of the infection. These results identify a novel way of apoptosis inhibition, involving only the most downstream modulator of mitochondrial apoptosis and suggest that *Chlamydia* has a protein dedicated to the inhibition of apoptosis to secure its survival in human cells.

## Introduction

Apoptosis plays a role in infections, and infectious agents, in many cases, interfere with host cell apoptosis pathways. This is best understood in viruses, which depend on the integrity of the infected cell and block apoptosis, often through expression of genes whose products resemble host anti-apoptotic proteins, such a Bcl-2-homologues [1]. Bacteria lack this ability to acquire host genes. However, especially intracellular bacterial pathogens have also developed ways of interfering with host cell signaling but this is mostly not understood molecularly [2].

Bacteria of the genus *Chlamydia* are obligate intracellular organisms that cause infections in humans and animals. *C. trachomatis* is the leading cause of bacterial sexually transmitted disease and of infectious blindness in the world [3]. *Chlamydia* targets numerous host cell pathways, acquires nutrients from the host cell and multiplies in a membrane-surrounded cytosolic vacuole in epithelial cells [4–6]. After about 2 days, the bacteria are released for the next cycle of infection [7]. As an obligate intracellular bacterium, *C. trachomatis* depends on host cell-integrity, and the experimental induction of apoptosis blocks its intracellular development [8]. *C. trachomatis*-infection has, however, strong anti-apoptotic activity, and *C. trachomatis*-infected human cells are remarkably resistant to apoptosis induced by external stimuli. It is likely that this activity is required to counteract the identifiable pro-apoptotic activity of the infection [9]. The genus *Chlamydia* encompasses a number of human and animal pathogenic species, and all five tested species can inhibit apoptosis [10–12].

Inhibition of apoptosis by chlamydial infection has been noted over 20 years ago [10] but its molecular mechanism is still unclear. Many studies are available that identify alterations of host cell signals propose that those changes contribute to chlamydial anti-apoptotic protection. As examples, pro-apoptotic Bad is sequestered by the chlamydial vacuole [13]; complexes of inhibitor of apoptosis proteins (IAPs) have been suggested to be required for protection [14]; interaction of the chlamydial protein TARP with the host cell adapter SHC1 has been proposed to drive pro-survival signals [15]; activation of ERK-signalling has been identified, which may reduce apoptosis-sensitivity [16, 17]; induction of HIF-1α [18] and down-regulation of p53 [19] have also been implicated.

While all of these mechanisms may have anti-apoptotic effects, it has to be noted that apoptosis-inhibition by *Chlamydia* is extremely powerful. Apoptosis of human cells can be triggered through either the death-receptor pathway or the mitochondrial [20]. *Ctr* inhibits specifically the mitochondrial pathway while signals that originate at death receptors and bypass mitochondria are not blocked [21]. *Ctr* inhibits all tested stimuli of mitochondrial apoptosis including staurosporine [10], etoposide [10], CD95-signals (in cells where a mitochondrial contribution is required) [10, 21], UV-light [22], and dsRNA [23]. These stimuli trigger diverse upstream pathways that converge on mitochondria: a crucial step of mitochondrial apoptosis is the release of cytochrome *c* [24], and *Ctr*-dependent inhibition of apoptosis blocks this release [10]. *Ctr*, therefore, either inhibits apoptosis separately in all upstream signaling pathways (i.e., inhibits signals that originate from the physical insult of UV-light, the pharmacological agent staurosporine and so on), or there is one major apoptosis-block downstream of a point where the various signals converge.

Of the mechanisms proposed for the *Ctr*-mediated anti-apoptotic effect, two act directly at mitochondria and may be able to explain the strong inhibition. First, the Bcl-2-homologue Mcl-1 is strongly upregulated during chlamydial infection [25]. High levels of Mcl-1 inhibit mitochondrial apoptosis profoundly, and this may therefore be an explanation for the broad apoptosis-inhibition by *Ctr*. Secondly, a loss of the pro-apoptotic Bcl-2-family member, the BH3-only protein Bim and other BH3-only proteins has been reported upon chlamydial infection [22, 26], although some of this may be an extraction artefact [27]. BH3-only proteins are essential triggers of mitochondrial apoptosis, and their loss may also explain profound protection against apoptosis during chlamydial infection. Another conceivable mechanism is an alteration in the retro-translocation of Bax and Bak from mitochondria [28].

Our understanding of mitochondrial apoptosis has much improved in recent years, and new tools have become available, especially small molecules inactivating individual anti-apoptotic Bcl-2-family proteins (“BH3-mimetics”) [29, 30]. This now permits the study of mitochondrial apoptosis and its inhibition by *C. trachomatis* in greater detail than had been possible previously. Here, we used a number of tools to map apoptosis-inhibition by *C. trachomatis*-infection. Genomic deletions of Bcl-2-family proteins, the use of Bcl-2-family inhibitors and of recombinant BH3-only proteins in cell-free systems show that current models of apoptosis-inhibition are unable to explain the chlamydial anti-apoptotic effect. We identify an interference with both effectors of mitochondrial apoptosis, Bax and Bak, as the point of inhibition by *Ctr*. Only one human protein is known to act at this level, VDAC2 [31]. We, therefore, included VDAC2 in our analysis. We further found that a chlamydial protein, the major outer membrane porin (OmpA/MOMP), when expressed in uninfected human cells, inserted into mitochondrial membranes. OmpA alone blocked apoptosis and reproduced the changes to Bak-activation observed during infection, reminiscent of the activity of VDAC2.

## Results

### Mcl-1 is not essential to Ctr-mediated inhibition of apoptosis

**Fig. 1:**
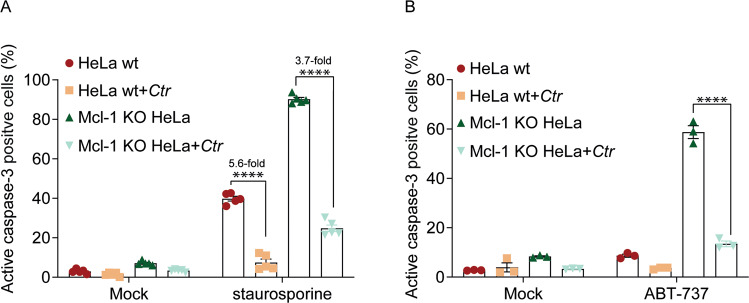
Ctr-infection inhibits apoptosis independently of Mcl-1. **A** HeLa wt or Mcl-1-deficient cells were infected with *Ctr* (MOI = 5) for 24 h. Cells were treated with 1 µM staurosporine for 5 h. Apoptosis was measured by staining for cells expressing active caspase-3. Data show means/SEM of five independent experiments. ****p < 0.0001, two-way Anova for multiple comparisons. **B** Wt and Mcl-1-deficient HeLa cells were infected with *Ctr* (MOI = 5) for 24 h. Cells were treated with 10 µM ABT-737 for 12 h. Apoptosis was measured by staining for cells expressing active caspase-3. Data show means/SEM of three independent experiments. ****p < 0.0001, two way Anova for multiple comparisons.

Mcl-1 is upregulated in human cells infected with *Ctr* [25] (Supplementary Figs. 1 and  2 show controls for the gene-modified cells), which may explain the protection against apoptosis (for easier reference, the interaction of the relevant proteins during mitochondrial apoptosis is shown in Supplementary Fig. 3). We deleted Mcl-1 in HeLa cells, the most commonly used cell for chlamydial infection. As expected, Mcl-1-loss increased the sensitivity of Hela cells to staurosporine-induced apoptosis. However, the strength of protection by *Ctr*-infection (measured as fold-reduction of cells undergoing apoptosis) was similar in the presence and absence of Mcl-1 (Fig. 1A). ABT-737 is an inhibitor of Bcl-2 and Bcl-X_L_, which can induce apoptosis in human cells but not in the presence of normal levels of Mcl-1 [32]. Accordingly, Mcl-1-deficient cells were very sensitive to ABT-737-induced apoptosis. *Ctr*-infection provided protection against ABT-737 in these cells (Fig. 1B). For comparison, we deleted a second anti-apoptotic Bcl-2-family protein, Bcl-w and also observed a sensitization to staurosporine-induced apoptosis upon its loss, and again *Ctr*-infection provided similar protection (Supplementary Fig. 4A) in gene-deficient and in wt cells. Bcl-X_L_ was increased in some experiments (Supplementary Fig. 2). We, therefore, deleted Bcl-X_L_ and found the expected enhanced sensitivity to the Mcl-1-inhibitor alone. Infection with *Ctr* provided profound protection against this treatment (Supplementary Fig. 4B). Mcl-1, Bcl-w and Bcl-X_L_ are, therefore, not essential to the inhibition of apoptosis by *Ctr*-infection.

### Ctr-infection blocks both Bax- and Bak-induced apoptosis downstream of anti-apoptotic Bcl-2-proteins

**Fig. 2:**
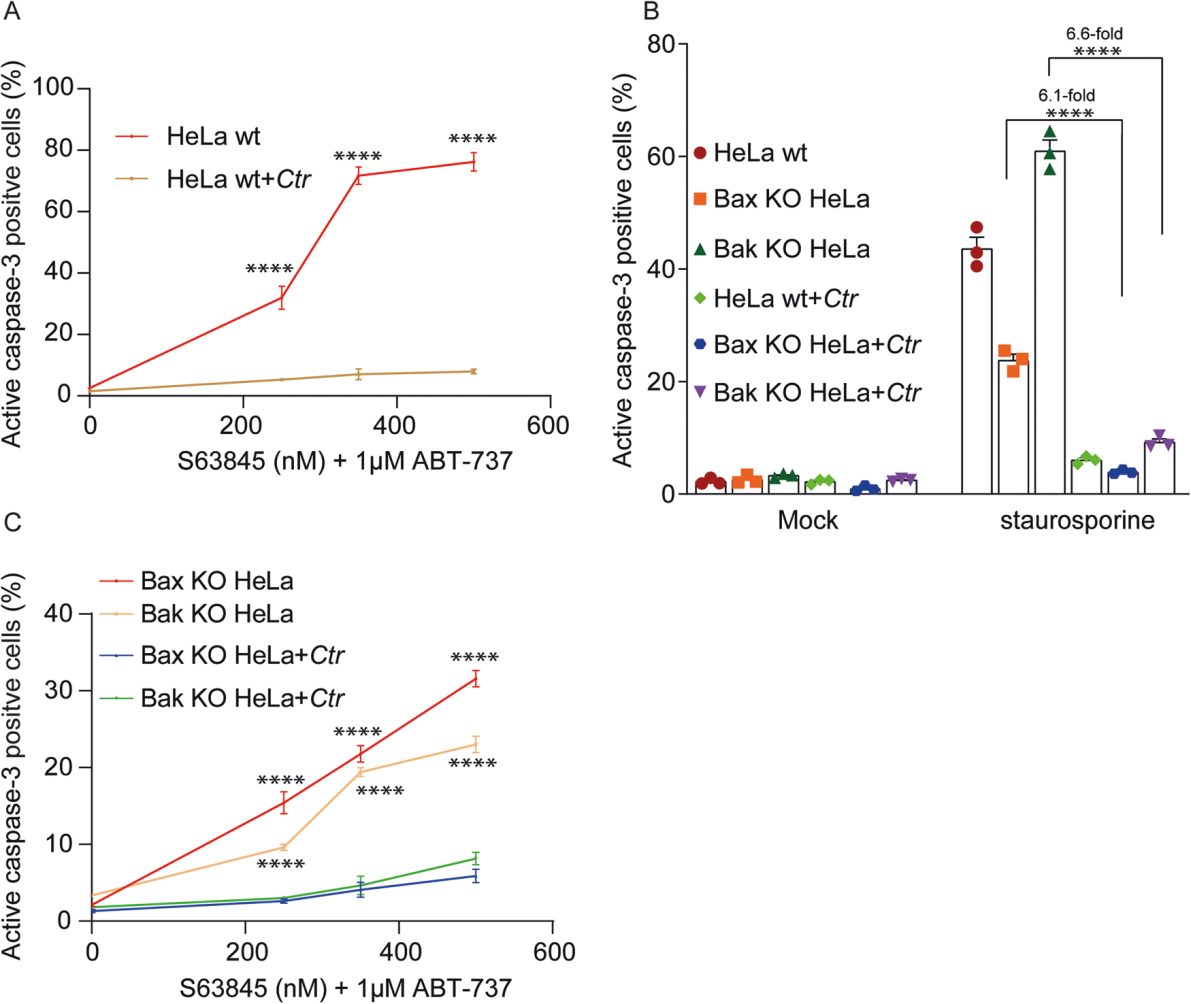
Ctr-infection inhibits both Bax- and Bak-dependent apoptosis downstream of anti-apoptotic Bcl-2-family proteins. **A**–**C** HeLa cell variants (wt, cells deficient in Bax or deficient in Bak) were infected with *Ctr* (MOI = 5) for 24 h or left uninfected. **A**, **C** cells were then treated for an additional 4 h with a fixed concentration of the Bcl-2/Bcl-X_L_-inhibitor ABT-737 (1 µM) and varying concentrations of the Mcl-1-inhibitor s63845. For (**B**) cells were treated with 1 µM staurosporine and collected after 5 h to measure apoptosis. All cells were fixed, stained for active caspase-3 and analyzed by flow cytometry. Data show means/SEM of three independent experiments. ****p < 0.0001 for infected vs. non-infected cells for each genotype (two way Anova for multiple comparisons).

In mitochondrial apoptosis, the activation of BH3-only proteins such as Bim causes the activation of the effectors of cytochrome *c*-release, Bax, and Bak. The anti-apoptotic Bcl-2-family proteins (Bcl-2, Bcl-X_L_, Bcl-w, Mcl-1, and A1) inhibit apoptosis by binding to BH3-only proteins and to Bax/Bak [20]. Small-molecule Bcl-2-family inhibitors shift the balance in favor of pro-apoptotic proteins and can induce apoptosis. We used ABT-737 in combination with the Mcl-1-specific inhibitor S63845 [30] to neutralize the major anti-apoptotic proteins. This combination induced apoptosis in wt HeLa cells, and this apoptosis induction was strongly inhibited by *Ctr*-infection (Fig. 2A). The inhibition of apoptosis by *Ctr*-infection, therefore, must lie downstream of the action of human Bcl-2-family proteins. Downstream of anti-apoptotic Bcl-2-proteins, the two proteins Bax and Bak are activated to release cytochrome *c*, which would ultimately lead to apoptosis. To test whether the activity of both Bax and Bak can be blocked by *Ctr*, we treated HeLa cells deficient in either gene with staurosporine or with ABT-737/S63845 and measured protection by *Ctr*-infection. Loss of Bax reduced staurosporine-sensitivity, while loss of Bak, curiously, increased it; loss of either protein reduced sensitivity to ABT-737/S63845. Infection with *Ctr* provided protection to a similar level in all cases (Fig. 2B, C). Because apoptosis in the absence of Bax is mediated by Bak and vice versa, *Ctr*-infection must be able to block both Bax and Bak activation or activity. We also tested a different apoptosis assay, namely the staining with annexin V/live-dead stain (because this assay requires unfixed cells and takes time, and apoptosis induced by Bcl-2-family inhibitors is very rapid, we used Bax-deficient cells). *Ctr*-infection blocked apoptosis also by this parameter (Supplementary Fig. 5A, B). To confirm the effect in a second cell line, we deleted Bax or Bak in the colon cancer cell line HCT116. As shown in Supplementary Fig. 5C, *Ctr*-infection blocked apoptosis induced by co-inhibition of Mcl-1 and Bcl-X_L_ (HCT116 cells show little sensitivity to ABT-737). Although in these cells apoptosis was almost exclusively mediated by Bak (Supplementary Fig. 5C), the *Ctr*-inhibitory effect on Bax-mediated apoptosis was very similar in both cell lines.

We considered the possibility that Drp1, a dynamin-like GTPase required for mitochondrial fission, is involved in chlamydial apoptosis inhibition. Some studies have reported that in the absence of Drp1, apoptotic cytochrome *c*-release is delayed (although others have not confirmed that [33]), and it has been reported that Drp1 is lost during *Ctr*-infection [34]. We confirmed the loss of Drp1 but found that Drp1 was not lost during infection with a chlamydial strain deficient in the protease CPAF, a strain that still has full anti-apoptotic activity [35] and whose mitochondria are protected against tBid-induced cytochrome *c*-release (Supplementary Fig. 6). A loss of Drp1 is therefore not involved in the protection against apoptosis upon chlamydial infection.

### Ctr-infection blocks the activation of Bax

**Fig. 3:**
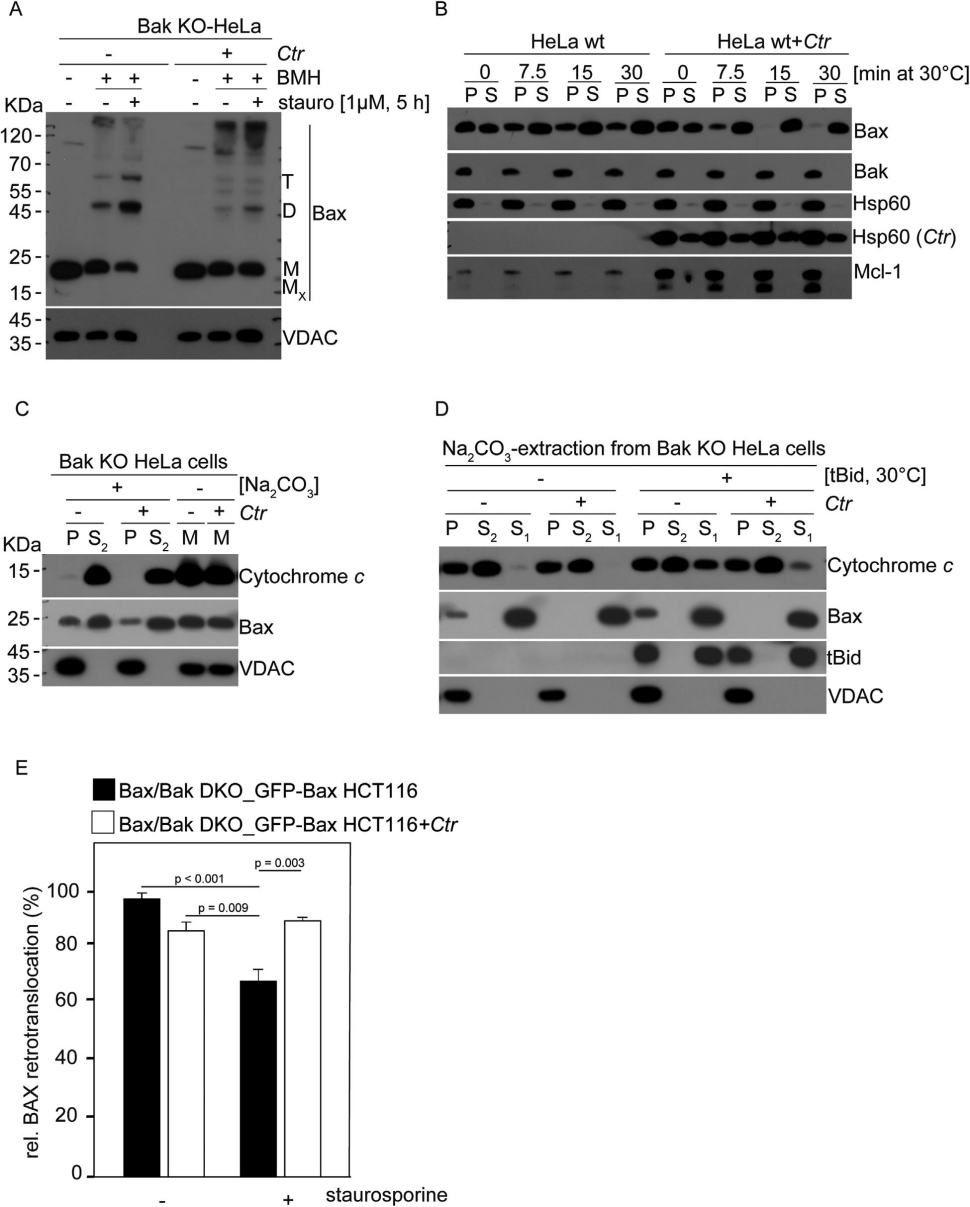
Ctr-infection inhibits membrane insertion and oligomerization of Bax in mitochondria. **A** Following treatment of *Ctr*-infected Bak KO cells with staurosporine (1 µM, 5 h), mitochondria were isolated, and same amounts of total protein were treated with the cross-linker bismaleimidohexane (BMH). Samples were analyzed for Bax by Western blotting and Hsp60 was used as loading control. Cross-linking demonstrated the presence of increased amounts of Bax-dimers upon staurosporine-treatment in non-infected cells but substantially less on mitochondria from *Ctr*-infected cells. Data are representative of three independent experiments. **B** Mitochondria were isolated from HeLa cells after 24 h infection with *Ctr* and incubated at 30 °C for the different times indicated to assess the diffusion of Bax from mitochondria. Supernatant (S) and pellet (P) fractions were obtained by centrifugation, immunoblotted and analyzed for Bax, Bak, Mcl-1, Hsp60, and chlamydial Hsp60 (*Ctr*). Data are representative of three independent experiments. **C** Mitochondria from either non-infected or *Ctr*-infected, Bak-deficient HeLa cells were isolated and directly subjected to sodium carbonate treatment to assess membrane-integration of Bax or boiled in Laemmli-buffer (M) directly to measure total Bax levels. After extraction, supernatants (S_2_) and pellets (P, not extracted, mostly integral membrane protein) were collected, and all samples were analyzed by Western blotting for membrane-bound proteins. Cytochrome *c* is a control for soluble protein released by the extraction treatment (detected only in supernatant) and VDACs is a control for membrane-bound proteins (detected mostly in pellet). Na_2_CO_3_, sodium carbonate extraction. Data are representative of three independent experiments. **D** Mitochondria were isolated as in (**C**) and incubated without or with recombinant tBid. Pellet and supernatant fractions were obtained by centrifugation, and supernatants (S_1_) were used to analyze tBid-mediated release of cytochrome *c*. Pellet fractions were then extracted by sodium carbonate to isolate integral membrane proteins (P) from extractable membrane proteins (S_2_). All protein fractions were run on SDS-PAGE and analyzed for indicated proteins. Data are representative of three independent experiments. **E** HCT116 cells deficient in Bax and Bak and stably expressing GFP-BAX were infected with *Ctr* or kept non-infected for 26 h, and then apoptosis was induced by staurosporine. Retrotranslocation of Bax was measured at all different conditions. Note, the unchanged rapid diffusion of Bax from mitochondria of *Ctr*-infected cells upon apoptosis induction. Data are representative of three different measurements. *P*-values according to one way Anova.

We then tested directly for a potential inhibition of the two Bcl-2-family effectors, starting with the analysis of Bax in Bak-deficient cells. Bax is mostly cytosolic or loosely attached to the outer mitochondrial membrane (OMM). Upon receipt of a pro-apoptotic stimulus, Bax-activation occurs through several steps: its accumulation at the OMM, its oligomerization, and its OMM-insertion, which drives the release of cytochrome *c* [33]. By chemical crosslinking we observed the predicted, apoptosis associated enhanced di-/trimerisation of Bax in cells treated with staurosporine. In *Ctr*-infected cells, we detected small amounts of different Bax-containing complexes but the staurosporine-induced di-/trimerisation of Bax was strongly reduced, and Bax remained mostly monomeric (Fig. 3A). We noticed no difference between the Bax levels on mitochondria from uninfected or *Ctr*-infected cells. However, Bax diffused rapidly away from mitochondria of *Ctr*-infected cells incubated in vitro (Fig. 3B), possibly indicating enhanced mitochondria-cytosol retro-translocation. Because this translocation is supported by anti-apoptotic Bcl-2-proteins [28] and Mcl-1 is strongly upregulated in *Ctr*-infected cells, we also tested Mcl-1-deficient cells but found a similarly accelerated diffusion of Bax (Supplementary Fig. 7A), suggesting that rapid diffusion of Bax from mitochondria upon *Ctr*-infection is Mcl-1-independent.

The insertion of Bcl-2-family proteins into the OMM can be tested by extraction of mitochondria with sodium-carbonate (NA_2_CO_3_; alkaline extraction) [36]. In this experiment, only membrane integrated proteins co-isolate with membranes whereas attached proteins are removed. The majority of Bax was loosely attached to mitochondria from uninfected cells as expected (fraction S2 in Fig. 3C) while a small amount remained membrane-associated during alkaline extraction (pellet fraction, P), indicating its insertion; in mitochondria from Ctr-infected cells, the inserted fraction was somewhat smaller (Fig. 3C). When the isolated mitochondria were incubated with recombinant tBid, a BH3-only protein that directly activates Bax [37], more Bax inserted into the membrane as expected (Fig. 3D). In mitochondria from *Ctr*-infected cells, Bax was completely removed by alkaline extraction, and tBid was unable to induce the insertion of Bax into the OMM (Fig. 3D). tBid was also reduced in its activity to release cytochrome *c* from mitochondria isolated from *Ctr*-infected cells (Fig. 3D); because this requires Bax or Bak, this was predicted by the failure of tBid to activate Bax. Finally, we tested Bax accumulation at mitochondria and found that while apoptosis induction with staurosporine reduced Bax-retro-translocation as expected in uninfected cells [28] *Ctr*-infection prevented this effect (Fig. 3E, Supplementary Fig. 7B–E), indicative of sustained retro-translocation of Bax in infected cells. Chlamydial infection thus causes a modification to host cell mitochondria that blocks the OMM-insertion of Bax and enhances its diffusion away from mitochondria.

### Ctr-infection interferes with the activation of Bak

**Fig. 4:**
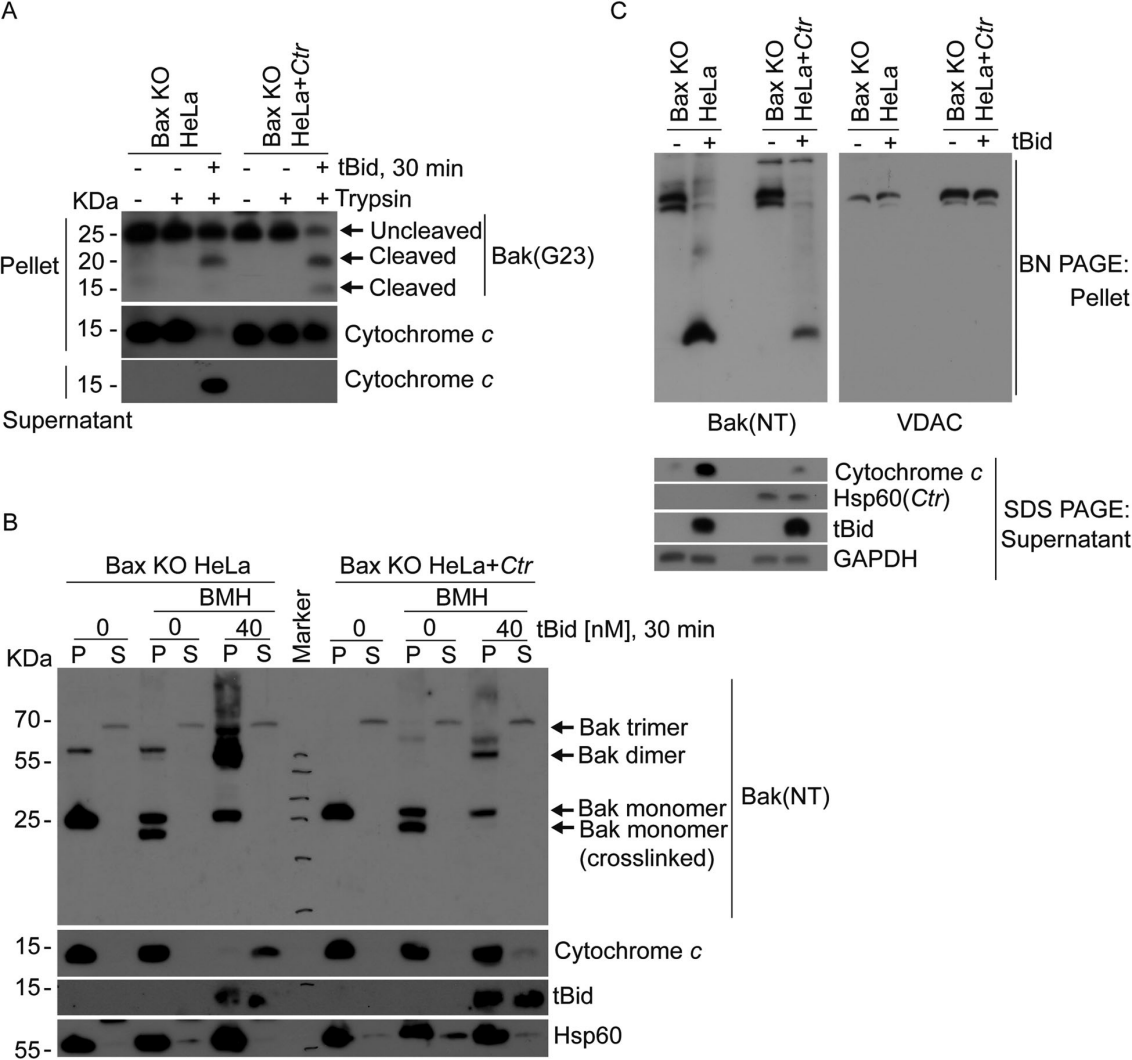
Ctr-infection prevents apoptosis-associated structural changes of Bak. **A** Mitochondrial fractions from uninfected or *Ctr*-infected Bax-deficient cells were incubated without or with trypsin to measure the enhanced Bak-protease sensitivity upon tBid-dependent Bak-activation. Intact Bak and its cleavage products were detected by immunoblotting for Bak (antibody G23; see Supplementary Table S1). To assess cytochrome *c* release, supernatants were analyzed separately. Note the enhanced trypsin-sensitivity of Bak on mitochondria from *Ctr*-infected cells. **B** Mitochondria were isolated and treated with tBid as in (**A**). In some reactions, proteins were crosslinked using the cysteine crosslinker BMH. To control for cytochrome *c*-release and Bak oligomerization, pellets (P) and released fractions (S) were analyzed separately by Western blotting for Bak, cytochrome *c*, tBid and mitochondrial Hsp60. Results are representative of three independent experiments. **C** Mitochondria isolated from uninfected or *Ctr*-infected, Bax-deficient HeLa cells were incubated with or without recombinant tBid for 30 min. Mitochondrial pellets were lysed and analyzed by BN-PAGE, followed by Western blotting for Bak and VDAC. Reaction supernatants were analyzed separately by SDS-PAGE to measure cytochrome *c*-release. Bak-containing complexes were visualized using anti-Bak (NT) antobodies; (see Supplementary Table S1). VDAC-containing complexes were detected using an antibody detecting all three VDAC isoforms. Note the tBid-induced formation of Bak-homodimers, which is strongly reduced on mitochondria from *Ctr*-infected cells (the forms of Bak identified in previous work [40] are indicated by the arrows). Data are representative of three independent experiments.

The activation of Bak can be monitored by a number of techniques. Bak is always inserted into the OMM and associated with the mitochondrial porin, voltage-dependent anion channel 2 (VDAC2). When activated, Bak is released from VDAC2, oligomerizes and exhibits increased sensitivity to trypsin-cleavage [38–40]. We used recombinant tBid on isolated mitochondria to activate Bak and to test the effect of *Ctr*-infection. We observed enhanced tBid-induced trypsin-sensitivity (Fig. 4A) as well as Calpain-sensitivity (Supplementary Fig. 8A) of Bak on mitochondria from *Ctr*-infected cells, suggesting a change in conformation. tBid-treatment induced the known Bak-containing complexes identified by cross-linking of mitochondrial proteins but these complexes were strongly reduced in abundance when mitochondria had been isolated from *Ctr*-infected cells (Fig. 4B). On native gels, Bak runs as a high molecular weight complex with VDAC2. When incubated with tBid, Bak was released from VDAC2 as expected and ran at a lower molecular weight form representing Bak-dimers [38, 39], (Fig. 4C, Supplementary Fig. 8B). However, when this assay was conducted with mitochondria isolated from *Ctr*-infected cells, formation of Bak-dimers upon tBid treatment was either strongly reduced or not detectable, depending on the choice of antibody (Fig. 4C, Supplementary Fig. 8B). The fraction of mitochondria-inserted (not mobilized by carbonate treatment) Bak remained unaltered (Supplementary Fig. 8C). This is most easily compatible with the existence of a mitochondrial factor in *Ctr*-infected cells that changes the conformation and alters the function of Bak.

### Ctr-infection causes the protection of mitochondria against BH3-only proteins in vitro

**Fig. 5:**
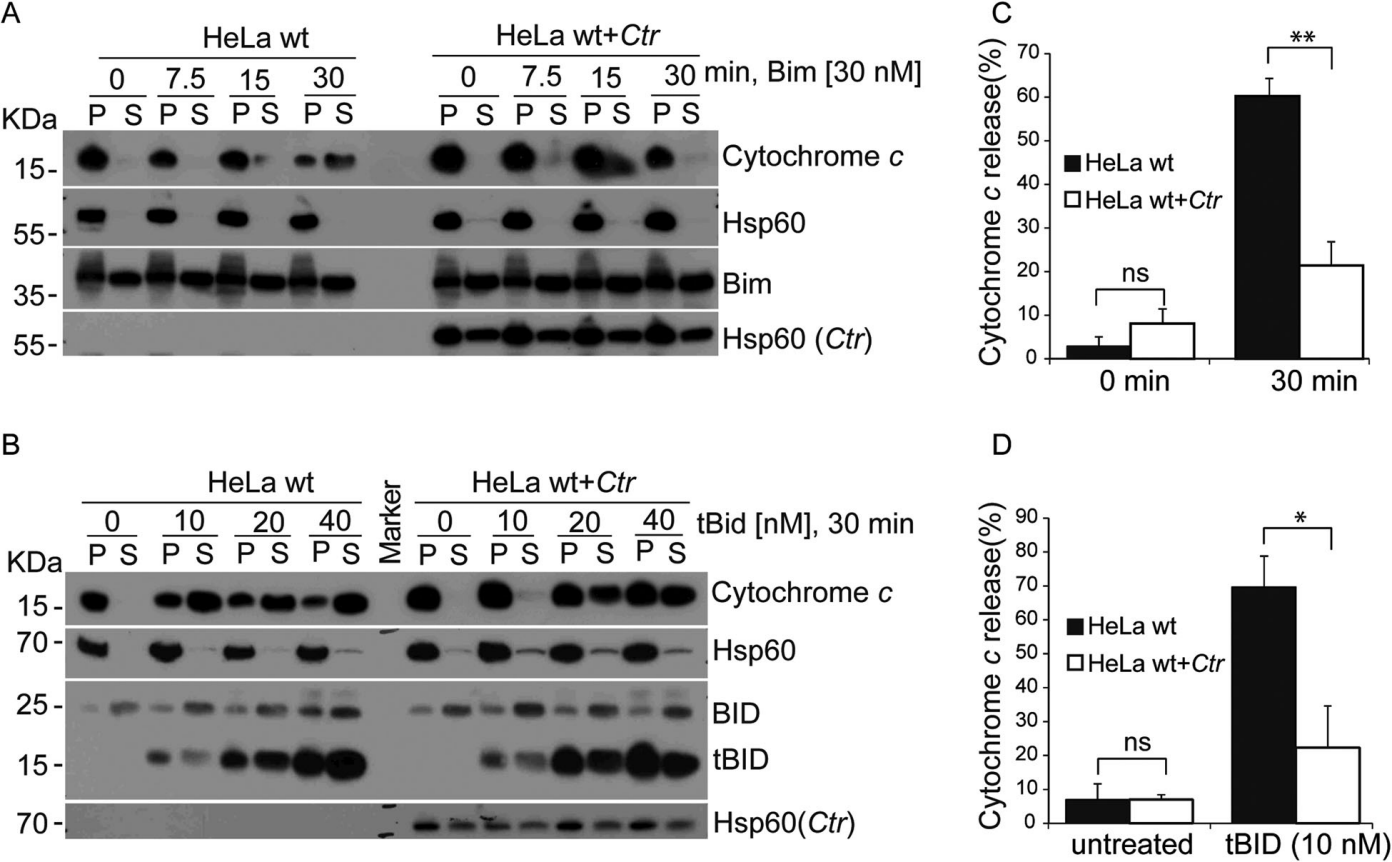
Ctr-infection protects mitochondria against the pro-apoptotic activity of BH3-only proteins. **A**, **B** Mitochondrial fractions were isolated from uninfected or *Ctr*-infected HeLa cells (MOI = 5, 24 h) and incubated for different lengths of time with 30 nM recombinant Bim (**A**) or with various concentrations of tBid for 30 min (**B**). Reactions were centrifuged to obtain pellet (P) and soluble (S) fractions, and fractions were analyzed by Western blotting for cytochrome *c* (to assess release into the S-fraction). Hsp60 is used as maker for mitochondria. Note the inhibition of cytochrome *c*-release from mitochondria isolated from *Ctr*-infected cells. The Western blot is representative of three independent experiments. **C**, **D** Quantification of release of cytochrome *c* from three experiments as shown in (**A**) and (**B**). ImageJ software was used to quantify cytochrome *c* release. Ratio of cytochrome *c* in the supernatant was calculated from its signal in the supernatant and the sum of cytochrome *c* in the supernatant and the pellet. Data are means/SEM of three independent experiments. **p < 0.01, *p < 0.05, (ns, not significant) *p* > 0.05, two-tailed unpaired t-test.

**Fig. 6:**
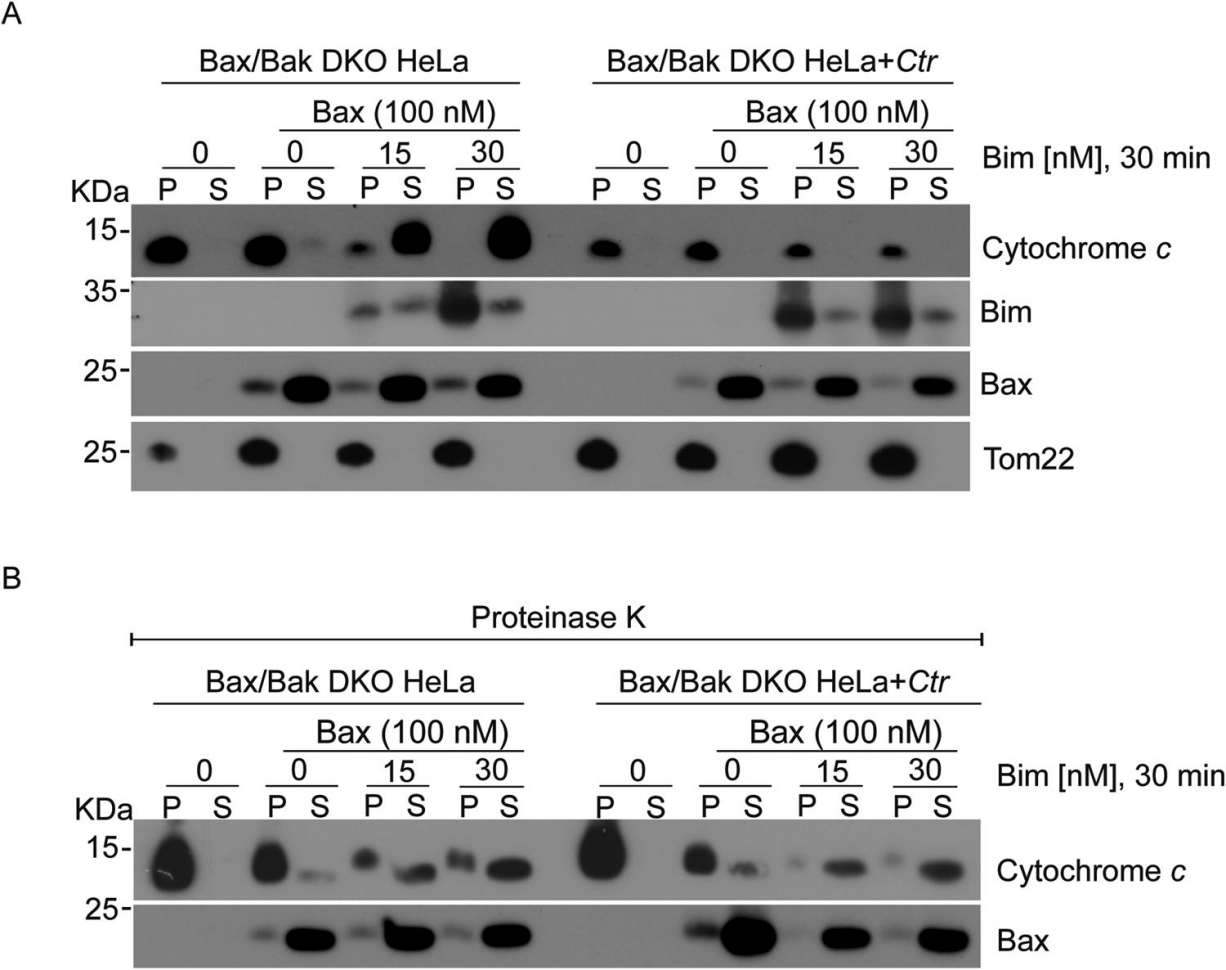
Ctr-infection generates a mitochondrial, proteinase K-sensitive factor that blocks release of cytochrome c. **A**, **B** Isolated mitochondria from Bax/Bak deficient HeLa cells (Bax/Bak-deficient), either *Ctr*-infected (MOI = 5, 24 h) or not infected were directly treated with a constant Bax concentration and various Bim concentrations (**A**), or digested with proteinase K prior to incubation with Bax and Bim (**B**). Samples were then collected, and release of cytochrome *c* was detected by Western blotting. Note the loss of inhibition of cytochrome *c*-release from mitochondria isolated from *Ctr*-infected cells upon proteinase K treatment.

During mitochondrial apoptosis, BH3-only proteins activate Bax and Bak, either through direct binding or through the inactivation of anti-apoptotic Bcl-2-proteins. As an aside to the above experiments, we had observed that the release of cytochrome *c* by tBid was reduced when mitochondria from *Ctr*-infected cells had been used. We used two important BH3-only proteins, tBid and Bim, to test the response of mitochondria from *Ctr*-infected cells more thoroughly. The results above suggested that Bax and Bak, which are necessary for cytochrome *c*-release, could not be activated by tBid on mitochondria from *Ctr*-infected cells. Indeed, the closer analysis showed that on mitochondria from wt HeLa cells (where both Bax and Bak are present to release cytochrome *c*), *Ctr*-infection blocked cytochrome *c*-release induced by either tBid or Bim (Fig. 5A–D). Similarly, when mitochondria from Bax/Bak-double-deficient cells were exposed to the combination of recombinant Bim and Bax, mitochondria released cytochrome *c* but this was blocked if the cells had been infected with *Ctr* (Fig. 6A). This inhibition required a proteinaceous component on the isolated mitochondria since digestion of mitochondria with proteinase K removed the protection (Fig. 6B). Although our above data suggested that such a protein was not a Bcl-2-family component (because *Ctr*-infection protected against Bcl-2-inhibitor treatment) we tested whether the association of anti-apoptotic Bcl-2-proteins with Bak would be altered upon infection. Staurosporine-treatment (which exposes the Bak-N-terminus) increased the efficiency of Bak-precipitation but we detected no increase in the association of Bak with the major anti-apoptotic Bcl-2-proteins (Supplementary Fig. 9A). Similar results were obtained when an antibody was used for IP that binds to Bak regardless of its activation status (Supplementary Fig. 9B).

**Fig. 7:**
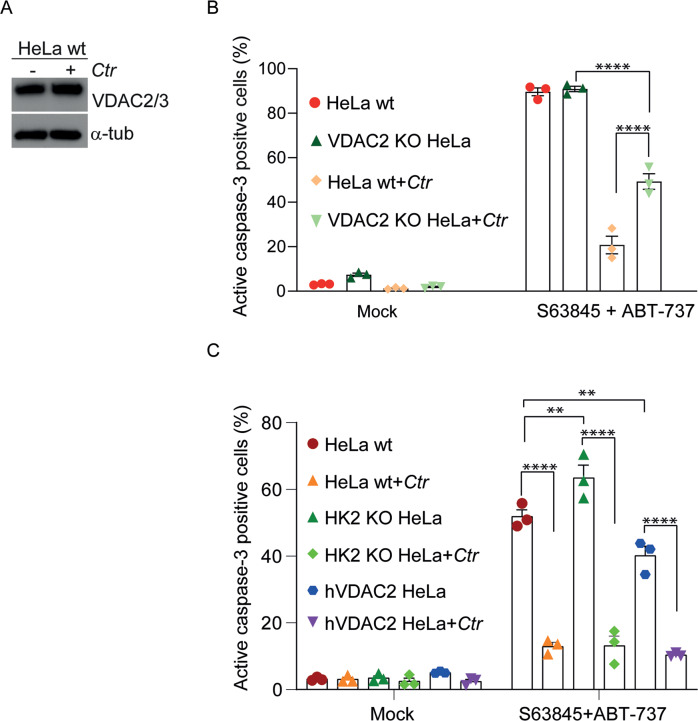
Loss of VDAC2 reduces but does not abolish protection against apoptosis by Ctr-infection. **A** Expression levels of VADC2/3 upon *Ctr*-infection. HeLa cells were infected with *Ctr*, and expression levels of VDAC2/3 were measured by Western blotting. No antibodies specific exclusively for VDAC2 are available. **B** HeLa wt or HeLa cells deficient in VDAC2 were infected with *Ctr* for 24 h and were then treated for an additional 4 h with the Bcl-2/Bcl-X_L_-inhibitor ABT-737 (1 µM) and the Mcl-1-inhibitor s63845 (500 nM). Apoptosis was measured by staining for cells containing active caspase-3. Data are means/SEM of three independent experiments. ****p < 0.0001, (ns, not significant) *p* > 0.05, two way Anova for multiple comparisons. **C** VDAC2-deficient HeLa cells reconstituted with human VDAC2 (hVDAC2), HeLa wt or HeLa cells lacking hexokinase 2 (HK2) were seeded and infected with *Ctr* for 24 h. Apoptosis was induced by the combination of ABT-737 (1 µM) and S63845 (500 nM) for 4 h and measured by flow cytometry for active caspase-3. Data are means/SEM of three independent experiments. ****p < 0.0001, **p *<* 0.01, (ns, not significant) *p* > 0.05, two way Anova for multiple comparisons.

The only known human protein that could perhaps explain the observed inhibition of Bax and Bak during *Ctr*-infection is the already mentioned porin VDAC2. VADC2 associates with Bak and plays a role in its import into the OMM [41]; VDAC2 can further serve as a platform of Bax-retro-translocation to the cytosol [42]. The mRNA-levels of VDAC2 were not significantly altered in a study measuring global gene expression during *Ctr*-infection [43]. We did however observe a slight up-regulation of VDAC2/3 protein levels in some experiments (Fig. 7A; the antibody cannot distinguish VDAC2 and VDAC3 proteins). To test for a role of VDAC2 in *Ctr*-mediated protection against apoptosis, we deleted the gene in HeLa cells. Intriguingly, although *Ctr*-infection was able also to protect VDAC2-deficient cells against apoptosis induced by ABT-737/S63845, this protection was clearly reduced compared to wt HeLa cells (Fig. 7B). The same was seen in cells with double deletions in VDAC2 and either Bax or Bak (Supplementary Fig. 10). It is noteworthy that deletion of VDAC2 strongly reduced Bak-levels (Supplementary Fig. 10A), as has been reported before in MEFs [44]; VDAC-deletion further reduced Bax-mitochondrial translocation [44], which likely explains the profound protection of VDAC2/Bax and Bak-double deficient Hela cells (Supplementary Fig. 10B, C).

Reconstitution of VDAC2-deficient cells with VDAC2 restored the protection by *Ctr*-infection (Fig. 7C). Hexokinase 2 has been suggested to be involved in protection by *Ctr*-infection [45]. However, hexokinase 2-deficient cells were also protected against apoptosis by the infection (Fig. 7C). *Ctr*-infection, therefore, can on the one hand protect against apoptosis downstream of all known host cell apoptosis regulators including VDAC2. On the other hand, VDAC2 clearly contributes to the full protection.

### Chlamydial OmpA can reproduce the molecular anti-apoptotic effect of chlamydial infection

**Fig. 8:**
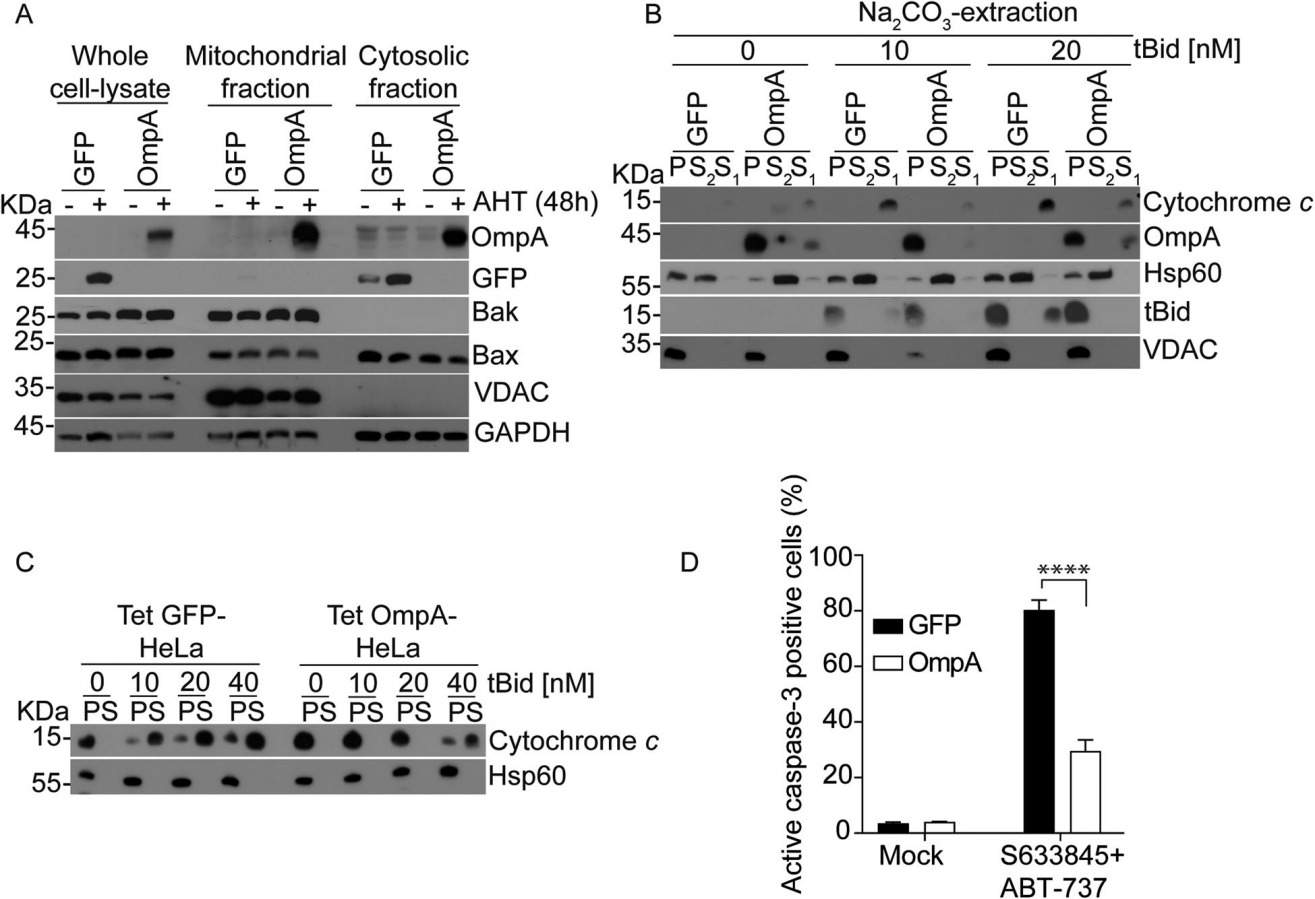
OmpA-expression inhibits mitochondrial apoptosis in HeLa cells, with OmpA inserting into mitochondrial membranes and blocking the tBid-induced release of cytochrome c. **A** HeLa cells carrying a construct encoding either OmpA (Tet OmpA) or GFP (Tet GFP) under the control of a tetracycline-inducible promoter were treated with anhydrotetracycline hydrochloride (AHT) to induce OmpA or GFP for 48 h. To analyze the subcellular localization of OmpA, cells were fractionated and mitochondrial and cytosolic fractions were subjected to Western blotting. Bak and VDAC were used as mitochondrial markers. **B** Mitochondria isolated from HeLa cells where OmpA or GFP had been induced for 48 h with AHT were incubated with recombinant tBid for 30 min as indicated. Mitochondria were pelleted, and supernatant fractions (labelled S1) were stored to assess tBid-induced release of cytochrome c. Mitochondrial pellets were resuspended in sodium carbonate buffer (pH 11.5) for protein-extraction. Membranes were then pelleted again (pellet fractions from this step are labelled P). Supernatants from this step (S2) were precipitated with trichloroacetic acid. Fractions were analyzed by Western blotting for cytochrome c (cyto c), OmpA, mitochondrial Hsp60, tBid, and VDAC. Note the retention of OmpA in the membrane (pellet) fraction. Less cytochrome c was released from OmpA-containing mitochondria compared to control (S1; no cytochrome c was seen in the precipitated S2-fractions, presumably as a matter of sensitivity). Data are representative of three independent experiments. **C** Mitochondria isolated from HeLa cells in which GFP or OmpA had been induced as above were incubated with various concentrations of recombinant tBid. Pellets (P) and supernatants (S) were collected and analyzed by Western blotting. Hsp60 was used to identify mitochondria. Note the reduced release of cytochrome c from OmpA-expressing mitochondria. Data are representative of three independent experiments. **D** Transgenic HeLa cells constitutively expressing GFP or OmpA from Ctr, were treated with ABT-737 (1 µM) and S63845 (500 nM) for 4 h. Cells were fixed, stained for active caspase-3 and analyzed by flow cytometry. Data show means/SEM of six independent experiments. ****p < 0.0001 two way anova multiple comparisons.

These results suggested that Bax and Bak, when apoptosis was induced, interacted with a mitochondrial protein that was generated or modified on mitochondria of infected cells. VDAC2 was involved, suggesting that the interaction of Bax/Bak with a mitochondrial porin plays a role. Porins are β-barrel proteins that regulate the flux of solutes across the outer membrane both in mitochondria and in Gram-negative bacteria. No bacterial porin with anti-apoptotic activity has been described. However, there is no argument against the possibility that indeed a bacterial porin may have similar activity as VDAC2 and may be involved in the inhibition of apoptosis by *Ctr*. *Ctr* has a main porin in its outer membrane, the major outer membrane porin (MOMP; we will here use the gene name, OmpA). We proceeded to test the possibility that OmpA may have anti-apoptotic activity and may be a candidate for the activity during infection. We generated a number of HeLa cell lines that carry the *Ctr ompA* gene. Inducibly expressed OmpA was to a substantial extent found in the mitochondrial fraction of HeLa cells (Fig. 8A). Mitochondrial isolation and carbonate extraction from OmpA-expressing HeLa cells showed tight association/integration of OmpA with mitochondrial membranes irrespective of tBid-treatment (Fig. 8B). tBid inserted normally in mitochondria from cells expressing OmpA (Fig. 8B), but OmpA-expression reduced mitochondrial sensitivity to tBid-induced cytochrome *c*-release, as we had observed for *Ctr*-infection (Fig. 8C). HeLa cells expressing OmpA constitutively (Fig. 8D, Supplementary Fig. 1H) were protected against apoptosis induced by inhibition of anti-apoptotic Bcl-2-proteins. Thus, OmpA indeed has anti-apoptotic activity that can block apoptosis downstream of anti-apoptotic Bcl-2 proteins, as we had found for *Ctr*-infection.

**Fig. 9:**
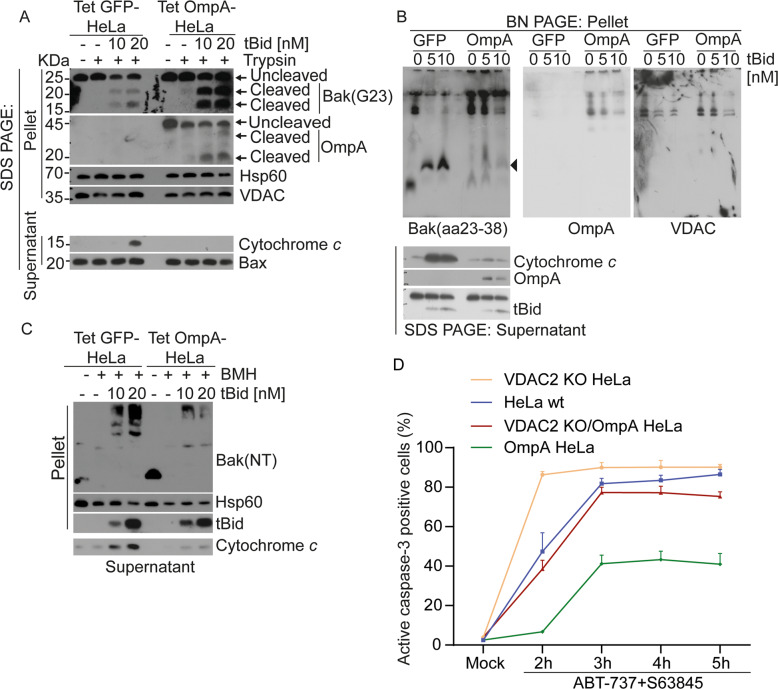
Ectopic expression of OmpA inhibits Bak-activation at the same step as Ctr-infection. **A** Mitochondrial fractions from control or OmpA-expressing cells were incubated without or with trypsin to identify the enhanced Bak-protease sensitivity upon its tBid-dependent activation. Intact Bak or OmpA with their cleavage products were detected by immunoblotting. To assess cytochrome *c* release supernatants were analyzed separately using a cytochrome *c* antibody. Note the trypsin sensitivity of OmpA upon tBid-treatment of mitochondria. GFP or OmpA was induced by treatment with anhydrotetracycline for 48 h. (representative of three independent experiments). **B** Mitochondria isolated from uninfected, FLAG-OmpA- or GFP-expressing HeLa cells were incubated with various concentrations of tBid for 30 min. Mitochondrial pellets were lysed and analyzed by BN-PAGE, followed by Western blotting for Bak, OmpA, and VDAC. Reaction supernatants were analyzed separately by SDS-PAGE to control for cytochrome *c*-release. Bak-containing complexes were visualized using antibody Bak(aa23-38); (see Supplementary Table S1). OmpA- and VDAC-containing complexes were detected using specific antibodies. The arrowhead indicates Bak-dimers. Data are representative of three independent experiments. **C** HeLa cells carrying tetracycline-inducible constructs were treated with AHT for 48 h to induce GFP or (untagged) OmpA. Mitochondria were isolated and treated with tBid as indicated. In some reactions, proteins were crosslinked using the cysteine crosslinker BMH. To control for cytochrome *c*-release and its inhibition by OmpA expression, released fractions (Supernatant) were analyzed separately by Western blotting for cytochrome *c*. Results are representative of three independent experiments. **D** After treatment of HeLa cell variants (Wt, cells deficient in VDAC2, cells constitutively expressing FLAG-OmpA or cells deficient in VADC2 and expressing FLAG-OmpA) with the combination of 1 µM ABT-737 and 0.5 µM S63845 for various times, active caspase-3 was determined by flow cytometry analysis. Data show means/SEM of three independent experiments.

We mapped the anti-apoptotic activity of OmpA further. We had already found that on mitochondria from *Ctr*-infected cells, Bak became more sensitive to trypsin cleavage when exposed to pro-apoptotic stimuli (Fig. 4A, above). We observed similar enhancement of trypsin-sensitivity of Bak on mitochondria from OmpA-expressing cells treated with tBid (Fig. 9A). Remarkably, this pro-apoptotic condition also enhanced the trypsin-sensitivity of OmpA itself (Fig. 9A). By BN-PAGE, upon activation through tBid, Bak was observed predominantly in a low, dimeric molecular weight form on mitochondria from uninfected cells but this fraction of free Bak was much reduced on mitochondria from OmpA-expressing cells (Fig. 9B). As shown above, when proteins are cross-linked on mitochondria during apoptosis, Bak forms large complexes, and this complex formation is reduced in *Ctr*-infected cells. A similar pattern of Bak-crosslinking was seen in uninfected cells expressing OmpA: substantially less Bak was detectable in large complexes when mitochondria were treated with tBid (Fig. 9C). OmpA, therefore, appears to be able to interfere with Bak activation, in a way indistinguishable from *Ctr*-infection.

Deletion of VDAC2 enhanced the sensitivity of HeLa cells to Bcl-2-family inhibitors as expected (Fig. 9D). To test whether OmpA could have the same anti-apoptotic effect as VDAC2, we reconstituted VDAC2-deficient cells with OmpA (Supplementary Fig. 1H). As hypothesized, this reversed the enhanced susceptibility of VDAC2-deficient cells (Fig. 9D), indicating that OmpA can indeed replace the anti-apoptotic function of VDAC2. Taken together, these results are strong evidence that *Ctr* inhibits apoptosis by mimicking the function of a human mitochondrial porin, and OmpA is a candidate for providing this activity.

## Discussion

In this study, we provide a detailed assessment of the molecular basis of the inhibition of apoptosis by *Ctr*. Our results show that the infection causes a block of apoptosis within the Bcl-2-family at mitochondria but downstream of anti-apoptotic proteins. Both Bax- and Bak-activation were inhibited: in the case of Bax, there was less background membrane-insertion and enhanced diffusion away from the membrane as well as sustained retro-translocation of Bax. tBid was unable to activate Bax on mitochondria from *Ctr*-infected cells. tBid can directly bind to and activate Bax [37]. The finding that it cannot perform this activity on mitochondria from infected cells suggests that the infection alters one of these two proteins or the OMM. Because the inhibition was lost upon protease digestion, it cannot be the membrane that is altered. Since we added an excess of recombinant tBid, and the Bax-containing complexes were altered on mitochondria from *Ctr*-infected cells, it seems very likely that Bax is the target of the chlamydial activity. Similarly, Bak-activation was blocked in the process. Exposure of the N-terminus was still possible (as suggested by the binding of the antibody for immune-precipitation), and Bak still appeared to dissociate from VDAC2 (as suggested by the native-gel experiments). The finding that Bak was less easily detectable upon this dissociation, its oligomerization was inhibited and its trypsin- and calpain-sensitivity increased also seems most easily compatible with its binding by a protein inhibitor. Intriguingly, the chlamydial porin OmpA reproduced the effect of *Ctr*-infection by all parameters tested. The results suggest that *Ctr* may exploit its evolutionary relationship with mitochondria to block apoptosis and to secure its intracellular growth.

β-barrel proteins are found both in bacterial and in mitochondrial outer membranes [46, 47]. OmpA is the major porin of the outer membrane of *Ctr*. It is in line with this similarity that OmpA blocks apoptosis, as we report here. The OmpA-association with mitochondrial membranes was alkaline-resistant, suggesting insertion into the membrane. Given the β-barrel structure of OmpA, it seems safe to suggest that the protein is probably inserted into the outer mitochondrial membrane, similar to mitochondrial porins.

The VDAC2-levels have previously been shown to regulate apoptosis: VDAC2 (but not VDAC1)-over-expressing cells are protected against Bak-dependent apoptosis [48], and VDAC2-deficient-thymocytes in mice die of spontaneous apoptosis [49]. Bak has been found in a complex with VDAC2 on mitochondria, which dissolved during induction of apoptosis [39] but this complex and its stability were, upon addition of tBid, altered in both *Ctr*-infected and OmpA-expression cells. The trypsin-sensitivity of OmpA changed when the cells were treated with tBid, suggesting that OmpA is in a complex targeted directly or indirectly by tBid. Bak-release from the VDAC2-containing complex was much less efficient in cells either infected with *Ctr* or expressing OmpA. OmpA may specifically sequester Bak upon its release from VDAC2. A simpler model is that VDAC2 and OmpA have the same molecular way of action, and the total level of VDAC2 + OmpA is what determines apoptosis sensitivity. This model is consistent with our observations that OmpA-expression can rescue Hela cells against ABT-737/S63845 and restores resistance to the wt level, and that over-expression of OmpA protects against apoptosis, while *Ctr*-infection has a reduced protective effect in cells lacking VDAC2.

While it seems clear that OmpA has the same activity as chlamydial infection and is, therefore, an excellent candidate for the anti-apoptotic factor of *Ctr*, this has not been shown directly. Our preliminary experiments have identified OmpA on mitochondria of infected cells (not shown). However, it will be substantial work to confirm this localization and to clarify how OmpA may translocate to mitochondria. We speculate that it may travel in outer membrane vesicles (OMVs). Gram-negative bacteria are known to release OMVs that may contain any type of cargo [50], and OMVs have been detected to be released from *Ctr* [51]. Furthermore, vesicles released from the inclusion and containing bacterial inclusion membrane proteins have been found by ultrastructural analyses outside the inclusion in the host cell, and OmpA has been specifically detected on host cell organelles (even though an association with mitochondria was not reported) [52]. In the case of *N. gonorrhoeae* it has recently been shown that porin (PorB) containing OMVs are released and PorB can integrate into macrophage mitochondria (where it had some pro-apoptotic activity) [53]. Given the high expression of OmpA on *Ctr*, it therefore seems likely that OmpA reaches the cytosol in substantial quantities, from where it may be inserted into the outer mitochondrial membrane, even though the precise mechanism is unclear. Genetic manipulation of *Chlamydia* is, although in principle now feasible, still very inefficient [54], and *ompA* is very likely an essential gene. Once we have understood better how OmpA interacts with mitochondrial proteins, it may be possible to replace OmpA with mutants that have an altered ability to interfere with apoptosis.

Our data map a *Ctr*-generated anti-apoptotic activity to direct interference with Bax and Bak and show that chlamydial OmpA can interfere with Bak activation, where it has an identical molecular activity to the one generated during infection. Although speculative, this is evidence for an intriguing concept: *Chlamydiae*, obligate intracellular bacteria, which have co-evolved with humans at least since the time when only protozoan eukaryotes existed [55], have used their relationship to the old eukaryotic symbionts, the mitochondria, to control apoptosis. Regulation of apoptosis is a major function of mitochondria. *Chlamydiae*, other intracellular organisms, can control mitochondrial apoptosis.

## Materials and methods

### Cell lines and cell culture conditions

HeLa 229 cervical carcinoma cells (Genome Reference Consortium, ATCC), HCT116 colorectal cancer cells, a gift from Dr. Christoph Borner, and 293FT cells (Invitrogen) were maintained in RPMI medium (Life Technologies, UK) supplemented with 10% FCS (tetracycline negative; Life Technologies). HCT116 Bax/Bak DKO cells stably expressing GFP-BAX [28] were cultured in antibiotic-free McCoy’s 5 A medium supplemented with 10% fetal bovine serum and 10 mM HEPES pH7.3. Cells were cultured in 5% CO2 at 37 °C in a humidified incubator.

### Constructs and generation of cell lines

In order to establish HeLa cells deficient in Bcl-X_L_, hexokinase 2, Mcl-1, Bcl-w, VDAC2, Bax, Bak or Bax and Bak, lentiviruses carrying gRNA targeting the respective genes were used. gRNAs were cloned into LentiCRISPR v2 (Addgene plasmid, #52961 [56]) or VDAC2 into LentiCRISPR v2 containing RFP instead of Puro als selectable marker. gRNAs sequences targeting Bcl-X_L_ ((5′-CACCGCAGGCGACGAGTTTGAACTG-3′), Hexokinase 2 (5′-CACCGGTCTACATAAGACCGTGCGG-3′), Mcl-1 (5′-CTCAAAAGAAACGCGGTAAT-3′), Bcl-w (5′-CACCGATGAGTTCGAGACCCGCTTC-3′), Bak (5′-ACGGCAGCTCGCCATCATCG-3′), Bax (5′-CAAGCGCATCGGGGACGAAC-3′) or VDAC2 (5′-CACCGCTACCTTCTCACCAAACACA-3′), were designed using the web-based sever (http://crispr.mit.edu/). VDAC2 KO HeLa cells expressing RFP as a selectable marker were generated by using gRNA sequences as described [44]. gRNA sequence (ATCGTTTCCGCTTAACGGCG) was used as non‐targeting control [9]. Following transduction target cells were selected using puromycin or by cell sorting (MoFlo Astrios cell sorter, Beckman Coulter) for high RFP-expression. hVDAC2 was reconstituted into VDAC2 KO HeLa cells expressing RFP using pEF1a plasmid with mutated gRNA (5′-ctataGGCCGAGGACTTGAGAGT-3′).

For the isolation of the gene coding for OmpA, HeLa cells were cultured in a 6-weel plate and infected with *Ctr* at a multiplicity of infection (MOI) of 5. After 24 h infected cells were collected and DNA (including chlamydial DNA) was extracted using the DNeasy blood and tissue Kit (Qiagen no. 69504) according to the manufacturer´s instructions. The OmpA ORF was amplified by PCR using the following primers: ((Flag-OmpA_del1-22-GW-Sense; 5′-CACCATGGATTACAAGGATGACGATGACAAGCTGCCTGTGGGGAATCC-3′ and OmpA-antisense; 5′-TTA GAA GCG GAA TTG TGC ATT TAC-3′) or (OmpA_del1-22-GW-Sense 5′-CACC ATG CTGCCTGTGGGGAATCC-3′ and OmpA-antisense; 5′-TTA GAA GCG GAA TTG TGC ATT TAC-3′)). The OmpA ORF (Gene ID: 5858320) was subsequently cloned into pENTR/SD/D-TOPO Gateway vector (Life Technologies) and was then inserted into the lentiviral vector pFCMVTO_GW_SV40_PURO_W [57] via Gateway LR recombinase reaction (Life Technologies). Lentiviruses were used to infect HeLa cells to establish cell lines stably expressing either FLAG OmpA or untagged OmpA. For inducible OmpA, HeLa cells were first transduced with lentivirus expressing Tet repressor as described [57], and selected cells were then infected with lentivirus carrying untagged OmpA. HeLa cells expressing either inducible or constitutive GFP were generated as above by cloning GFP into pFCMVTO_GW_SV40_PURO_W. Cells stably carrying the construct were selected with 1 μg/ml of puromycin (InvivoGen, #ant-pr-1).

### Lentivirus production and bacterial infection

For lentivirus production, 293FT cells were transfected with corresponding vectors using FuGene HD transfection (Promega, USA), following the manufacturer’s instructions. Packaging vectors were psPAX.2 and psMD2.G (Addgene Plasmids, #12260 and #12259; Dr Didier Trono). Virus-containing supernatant was collected, filtered and incubated with HeLa cells in the presence of 1 μg/ml of polybrene (Millipore, #TR-1003-G).

*Chlamydia trachomatis* serovar L2 (*Ctr*) was obtained from the American Type Culture Collection and propagated in HeLa cells. *Ctr* strains deficient in expression of chlamydial protease/proteasome-like activity factor (CPAF) (*Rst17*) and the isogenic, CPAF competent strain (*Rst5*) [35] were a kind gift from Dr Raphael Valdivia (Duke University School of Medicine, Durham, North Carolina, USA). Bacteria were purified over a Gastrografin density gradient (Bayer Vital, Leverkusen), followed by titration on HeLa cells and stored in SPG medium (0.2 M sucrose, 8.6 mM Na2HPO4, 3.8 mM KH2PO4, 5 mM glutamic acid [pH 7.4]) at −80 °C. Fresh aliquots were thawed for each experiment. Cells were infected at a multiplicity of infection (MOI) of 5 in complete culture medium.

### Analysis of apoptosis by flow cytometry

Apoptosis was induced by treatment with either staurosporine (Sigma, #S4400), ABT-737 (Selleck Chemicals, #S1002), Mcl-1-inhibitor S63845 (APExBio # A8737), the combination of ABT-737 and the Mcl-1-inhibitor S63845, or the combination of Bcl-X_L_ inhibitor A‑1155463 (Biozol, #S7800) and the Mcl-1-inhibitor S63845. Cells were collected, washed and fixed with 4% paraformaldehyde (Morphisto, #11762.00250) for 30 min at room temperature, followed by staining with anti-active caspase-3 antibody (BD Pharmingen, #559565) in PBS (Life Technologies, #14190169) containing 0.5% Saponin (Roth, #4185.1) and 0.5% bovine serum albumin (BIOMOL, #BSA-50). Alexa Fluor 647-conjugated donkey anti-rabbit IgG (Dianova, #711605152) or Alexa Fluor 488 conjugated donkey anti-rabbit IgG (Dianova, #711-545-152) were used as secondary antibody and cells were analyzed by flow cytometry using a FACS Calibur Flow Cytometer (Becton-Dickinson, Heidelberg), in combination with the software FlowJo version 10.4.

### Annexin V and LIVE/DEAD staining

Bax KO HeLa cells were seeded und infected with *Ctr* at a MOI of 5. 24 h post-infection, apoptosis was induced with the combination of S63845 (0.5 µM) and ABT-737 (1 µM) for 4 h. After trypsinization, cells were collected and wash in annexin binding-buffer, then incubated with Annexin V-FITC (Invitrogen, #BMS306FI-300) and Live Dead Fixable Far Red (Life Technologies, #L10120) for 30 min on ice. Prior to adding of 2% PFA, cells were washed twice with binding-buffer, and then fixed for 15 min on ice, followed by two washing steps. Cells were analysed by flow cytometry using FACS Calibur Flow cytometry.

### Mitochondria isolation and cytochrome c release assays

Cells were harvested, washed and resuspended in MB-EDTA buffer. Mitochondria were isolated by passing cells through a 27 G needle using 1 mL syringe as described [36]. For cytochrome *c* release assays, mitochondria were pelleted and resuspended in mitochondrial experimental buffer (125 mM KCl, 10 mM HEPES [pH 7.4], 5 mM KH2PO4, 0.5 mM EGTA, 4 mM MgCl2), followed by incubation with either recombinant Bim [58], recombinant tBid (a kind gift from Dr Jean-Claude Martinou, Geneva), recombinant Bax [36] or the combination of recombinant Bax and Bim at 30 °C. After centrifugation at 10,000xg for 10 min, pellet and supernatant fractions were boiled in Laemmli-buffer at 95 °C and analyzed by Western blotting.

### Subcellular fractionation to analyze OmpA subcellular localization

HeLa cells inducibly expressing either GFP or OmpA were incubated without or with 100 nM anhydrotetracycline hydrochloride (AHT) (IBA Life Sciences, #2-0401-001) for 48 h to induce expression. Mitochondrial fractions were isolated as described above and supernatants were centrifuged for 60 min at 4 °C and 120,000 x g. The resulting supernatants (cytosolic fractions) together with mitochondrial fractions were subjected to immunoblotting using VDAC and GAPDH as marker proteins for cytosolic and mitochondrial fractions.

### Protease digestion of mitochondria using proteinase K

Mitochondria were resuspended in mitochondrial experimental buffer containing 10 mg/mL proteinase K (Carl Roth, #7528.1) and incubated for 10 min on ice. The reaction was quenched by adding phenylmethanesulfonyl fluoride (PMSF) (Sigma, #93482) [59]. Following centrifugation, pellet fractions were washed and used for cytochrome *c* release assays.

### Analysis of Bax or Bak oligomerization using the chemical cross-linker bismaleimidohexane

Formation of Bax-dimers or Bak high-molecular-weight complexes was analyzed using bismaleimidohexane (BMH) (Thermo Fisher Scientific, #22330) as described [60]. Briefly, mitochondrial pellets either isolated from staurosporine-treated Bak KO HeLa cells, ABT-737 + s63845-treated Bax KO HeLa cells or upon tBid-treatment were resuspended in crosslinking buffer (20 mM HEPES [pH 7.5], 100 mM sucrose, 2.5 mM MgCl2, and 50 mM KCl) and were then treated with 0.5 mM BMH for 30 min at room temperature in the dark. Crosslinking was stopped by adding 0.5 mM dithiothreitol (DTT) (Panreac AppliChem, #A2948.0025), and samples were heated at 95 °C for 5 min and analyzed by Western blotting.

### Assessment of Bak conformational change by limited trypsin proteolysis

Mitochondria were incubated in mitochondrial experimental buffer containing 100 µg/mL trypsin (Life Technologies, #25300096) for 20 min on ice as described [60]. Protease activity was stopped by adding 1 mM PMSF and samples were characterized by Western blotting using antibody directed against Bak BH3 domain (Bak(G23)) (Santa Cruz, #sc-832).

### Assessment of Bak conformational change by limited calpain proteolysis

Bak-limited proteolysis by Calpain was performed as described [61]. Briefly, mitochondria were centrifuged following the apoptotic stimulus with recombinant tBID, and resuspended in calpain assay buffer. Reactions were then incubated with 20 nM calpain (United States Biological, #370550) containing 0.5 mM CaCl_2_ (Sigma-Aldrich, #C5670-100G) for 30 min at room temperature and samples were boiled in Laemmli sample Buffer for 10 min. Western Blot was performed and Bak was analyzed with anti-Bak(G23) antibody (Santa Cruz Biotechnology, # sc-832).

### Mitochondria isolation and Bax-diffusion assays

HeLa cells were either non-infected or infected with *Ctr* for 24 h at a MOI of 5. Mitochondrial fractions were isolated as described above, followed by incubation at 30 °C at different time points and samples were centrifuged for 10 min at 4 °C and 10,000xg. The resulting supernatants (release fractions) together with mitochondrial fractions were subjected to immunoblotting using Hsp60 and Hsp60_*Ctr* as marker proteins for mitochondrial fractions and infection.

### Determination of membrane insertion using sodium carbonate extraction

Isolated mitochondria that had been treated without or with tBid were resuspended in 0.1 M sodium carbonate (pH 11.5) (Sigma, #S7795) and incubated at 4 °C for 30 min [60]. The suspensions were centrifuged at 4 °C for 60 min at 125,000 x g in Thinwall Polyallomer Tubes (Beckman Coulter, #331372) in a TLA 110 rotor and pellet fractions (P) (membrane inserted proteins) were collected, resuspended and heated in Laemmli buffer containing DTT. Supernatants were incubated in 12.5% tri-chloroacetic acid (TCA) for 30 min on ice, followed by centrifugation for 60 min at 150,000 x g at 4 °C to precipitate proteins. Pellets were washed with acetone (Carl Roth, #9372.1), dried for 5 min at room temperature, resuspended and heated in Laemmli buffer containing DTT. Fractions were loaded onto the 12.5% SDS gel for Western blotting.

### Bak immunoprecipitation

For *Ctr*-infected cells, Bak protein was immunoprecipitated according to the protocols described in [62]. For IP non-activated Bak, anti-Bak(7D10) (a kind gift from Dr. David Huang, WEHI) was used, and cells were harvested and directly lysed in buffer containing 1% CHAPS (Carl Roth, #1479.2). For IP of activated Bak, cells were treated as indicated the presence of caspase inhibitor QVD-OPh (Gentaur (ApexBio), #GEN2269261) (10 µM). Cells were then harvested and subsequently lysed in buffer containing 1% CHAPS as reported [62]. Following centrifugation at 13,000xg, protein concentration of the supernatants was quantified using Bradford assay (Bio-Rad) and equal amounts of protein were precleared with protein G agarose beads (Roche, #11719416001) for 60 min at 4 °C with constant agitation. Precleared lysates were incubated with Bak antibodies (either Bak(aa23-38) or Bak(Ab-1)) or with Bak(7D10) for 120 min at 4 °C, followed by incubating with protein G agarose beads (Millipore Sigma, #11719416001) for an additional 60 min under constant agitation to allow the antibodies to bind to the beads and samples were collected and separated by SDS-PAGE and immunoblotted.

### Blue native-PAGE and western blotting

Mitochondrial pellet fractions were lysed in buffer containing 1% digitonin (Calbiochem, #300410) [63]. After 30 min on ice, samples were centrifuged and the supernatants were run on a 6–16.5% polyacrylamide gradient-gel at 4 °C, followed by transfer on PVDF membranes (GE Healthcare, #10600023). Bak antibodies used were Bak(aa23-38) and Bak(NT) (Millipore, #06-536). For detection of VADC, anti-VDAC (Cell Signalling, #4661) was used. Signals were detected using horseradish peroxidase-conjugated secondary antibodies (see below) and enhanced chemiluminescence (GE Healthcare or Thermo Scientific).

### SDS-PAGE and western blotting

Cells were lysed either in 8 M Urea or in RIPA buffer (Thermo Fisher, #89900) and boiled in Laemmli-buffer containing DTT. Samples were heated for 5 min. Antibodies used were: Bak(NT), Bak(aa23-38), Bak(Ab-1), Bak(G23) as above. The following antibodies were purchased from Cell Signaling unless indicated otherwise: anti-Bim (# C34C5), anti-GAPDH (Millipore, #MAB374), anti-Bcl-X_L_ (#54H6), anti-Bcl-2 (#2870), anti-Bax (#2772), anti-VDAC (#4661), anti-Hsp60 (#4870), anti-Mcl-1 (BD, #559027), anti-Hsp60(*Ctr*) (Enzo Life Sciences, #ALX-804-072), anti-Tom22 (Santa Ccruz, sc-58308), anti-Bcl-w (#2724 S), anti-GFP (Roche, #11814460001), anti-cytochrome *c* (#11940) and anti-tBid [64], anti-BiP (Cell signalling, #3177), anti-VDAC2/3 (Thermo Fisher, #PA141205). Peroxidase-conjugated secondary antibodies were goat anti-rabbit IgG (Sigma, #A6667), goat anti-rabbit Fc (Sigma, #AP156P), goat anti-mouse IgG (Dianova, #115035166), goat anti-mouse Fc (Sigma, #AP127P) and goat anti-rat IgG (Dianova, #112035062).

### Fluorescence loss in photobleaching measurements

Cells were seeded on a Nunc Lab-Tek Chambered Coverglass (VWR) at a density of 120,000 cells per chamber and incubated in McCoy’s 5 A medium, then infected with Chlamydia trachomatis serovar L2 (*Ctr*) for 26 h at a MOI of 5. Fresh aliquots of *Ctr* were thawed for each experiment. Infected and non-infected cells were treated with 1 µM staurosporine and 10 µM Q-VD-Oph for 2 h prior to the measurements.

Fluorescence loss in photobleaching (FLIP) experiments were performed in triplicate. Cells were imaged prior to bleaching using a Leica TCS SP8 DMi8 inverted, high-speed confocal microscope equipped with a PMT detector and a 63x/1,20 Plan-Apochromat W lens. A single spot in the analyzed cell was repeatedly bleached with 2 frames at 10 Hz using a 488 nm laser line (70% output). After each bleaching cycle, a picture was collected. One FLIP experiment was completed after nine cycles of repeated bleaching and image acquisition. The Leica Application Suite X software platform was used to analyze the fluorescence loss on the mitochondria of the selected cell. Additionally, an unbleached control cell was monitored during the measurement and used to exclude any photobleaching events that may occur during image acquisition.

## Supplementary information

Supplementary Figure and Table legends

Figure S1

Figure S2

Figure S3

Figure S4

Figure S5

Figure S6

Figure S7_1

Figure S7_2

Figure S8

Figure S9

Figure S10

Table S1

Pre-Authorship form

Uncropped original western blots files

Uncropped original western blots files

Uncropped original western blots files

Uncropped original western blots files

Uncropped original western blots files

Uncropped original western blots files

## Data Availability

The datasets generated during and/or analyzed during the current study are available from the corresponding author on reasonable request.
