# Supplementary material for: Chlamydia trachomatis inhibits apoptosis in infected cells by targeting the pro-apoptotic proteins Bax and Bak
Source: Cell Death Differ. 2022 Apr 9;29(10):2046–59. doi: 10.1038/s41418-022-00995-0 (PMC9525694; doi:10.1038/s41418-022-00995-0)
Supplement: Supplementary file 1 — Supplementary Figure and Table legends [file 41418_2022_995_MOESM1_ESM.docx]

**Figure/Table legends**

**Figure legends**

**Figure S1:** ***Genomic deletions in HeLa cells via CRISPR/Cas9 system***

**A** – **F**. HeLa cells (wt, Hexokinase 2 (HK2), Bcl-X_L_, Mcl-1-, Bcl-w-, VDAC2-, Bax-, Bak- or Bax/Bak-deficient as indicated) were lysed in RIPA buffer and deletion-efficiency was tested by Western blotting using the corresponding antibodies. α-tub (α-tubulin)

**G**. VDAC2 KO HeLa cells in (**C**) reconstituted with hVDAC2 were analyzed by Western Blot and immunoblotted for VDAC2 or α-tub as loading control.

**H**. VDAC2-deficient HeLa cells stably expressing FLAG-OmpA were generated and analyzed for expression of OmpA by immunoblotting for the proteins indicated, alongside HeLa cells carrying a non-targeting gRNA (H3), HeLa cells expressing FLAG-OmpA and the VDAC2-deficient maternal HeLa cells.

**Figure S2:** ***Protein-levels and subcellular localization of Bcl-2-family proteins during* Ctr*-infection***

**A**. HeLa cells were infected with *Ctr* (MOI=5) and lysed in 8 M urea after 24 h. Whole cell-lysates were run on SDS-PAGE, followed by Western blotting for the proteins indicated. Note the up-regulation of anti-apoptotic Mcl-1 and the loss of the pro-apoptotic BH3-only protein Bim. Data are representative of three independent experiments.

**B**. Mitochondrial and cytosolic fractions of HeLa cells were obtained by centrifugation. Isolated mitochondrial fractions were resuspended in KCl-buffer and incubated at 30°C for 30 min. Pellet (P) and supernatant (S) were separated after centrifugation, and all fractions were analyzed by Western blotting for indicated proteins. Data are representative of three independent experiments.

**Figure S3: *Schematic representation of mitochondrial apoptotic pathway***

Upon an apoptotic stimulus, BH3-only proteins (such as Bim or tBid) activate Bax and Bak (partly through direct interaction), which is followed by Bax/Bak-oligomerization change in membrane insertion and permeabilization of the outer mitochondrial membrane, leading to the release of cytochrome *c*. The interaction of pro-survival proteins (such as Mcl-1, Bcl-2 or Bcl-X_L_) with BH3-only proteins or with Bax/Bak prevents Bax- and Bak-mediated cytochrome *c* release to the cytosol. The combination of the inhibitors of Bcl-2/Bcl-XL and Mcl-1 (ABT-737/S63845) activates the system. Bcl-X_L_: B-cell lymphoma extra-large; Mcl-1: myeloid cell leukemia 1; tBid: truncated BH3-interacting domain death agonist; Bcl-2: B-cell lymphoma 2, Bim: Bcl-2 interacting mediator of cell death, Bax: Bcl-2-associated X protein, Bak: Bcl2-antagonist/killer.

**Figure S4: Ctr*-infection inhibits mitochondrial apoptosis in Bcl-w- and Bcl-X_L_-deficient cells***

**A**. HeLa cells (wt, or Bcl-w-deficient) were infected for 24 h with *Ctr* (MOI=5) or left uninfected. Cells were treated for 5 h with staurosporine (1 µM) as indicated. Cells were fixed, stained for active caspase-3 and analyzed by flow cytometry. Data show means/SEM of three independent experiments. ****, p ˂ 0.0001, ***, p ˂ 0.001, two-way Anova for multiple comparisons.

**B**. HeLa cells (wt or Bcl-X_L_-deficient cells) were infected with *Ctr* for 24 h and treated with the Mcl-1 inhibitor S63845 (100 nM) for 4 h. Apoptosis was assessed by active caspase-3 staining. Data show means/SEM of three independent experiments. ****, p ˂ 0.0001, ns (not significant), p ˃ 0.5, two-way Anova for multiple comparisons.

**Figure S5:  *Apoptosis measured by active caspase-3 staining correlates well with Annexin V/LiveDead-based markers of viability, and protection by* Ctr*-infection is not cell-type-specific***

**A**, **B**. HeLa cells wt or Bax-deficient were infected for 24 h and treated with the combination of the inhibitors of Bcl-2/Bcl-XL (ABT-737, 1 µM) and Mcl-1 (S63845, 0.5 µM) for an additional 4 h. Cell viability was assessed by Annexin V-staining and Live/Dead exclusion. Shown are (A) single dot plots representative of three different experiments and (B) are mean/SEM of three independent experiments.

**C**. wt, Bax- or Bak-deficient HCT116 cells were seeded and infected with *Ctr*. 24 h post-infection, cells were treated with A-1155463 (Bcl-X_L_-inhibitor, 100 nM) and S63845 (Mcl-1-inhibitor, 250 nM), and apoptosis was measured by flow cytometry with active caspase-3 staining. Data show means/SEM of three independent experiments. ****, p ˂ 0.0001, two-way Anova for multiple comparisons.

**Figure S6:**  ***Loss of Drp1 during infection but not the inhibition of apoptosis by* Ctr *depends on the chlamydial protease CPAF***

**A**. HeLa cells were infected with the *Ctr* L2-strain used for all other experiments here (*Ctr*), or with the mostly isogenic L2-strains Rst5 (CPAF-positive) or with Rst17 (CPAF-negative). After 24 h, cells were treated for 5 h with staurosporine (1 µM). Apoptosis was measured as the percentage of cells positive for active caspase-3. Data show means/SEM of three independent experiments. ****, p ˂ 0.0001, ns (not significant), p ≥ 0.5, two-way Anova for multiple comparisons.

**B**. HeLa cells were infected with the *Ctr* L2-strains (MOI=5) Rst17 (CPAF-deficient) or Rst5 (CPAF-proficient). After 24 h cells were lysed in RIPA buffer and incubated at 30°C with various time periods. Samples were boiled at 95°C in Laemmli buffer and subjected to Western blotting. Detection of Drp1 and GAPDH is shown. Hsp60(*Ctr*) served as infection control. The immunoblots are representative of three independent experiments.

**C**. HeLa cells were infected with the Rst17 (CPAF-deficient) or Rst5 (CPAF-proficient) strains of *Ctr*. 24 h post-infection, mitochondria were isolated and incubated at 30°C for 30 min with various concentrations of tBid. Supernatants (S) and pellets (P) were obtained by centrifugation, and samples collected were subjected to Western blotting. Detection of Drp1, cytochrome *c*, tBid and Hsp60 is shown. Chlamydial Hsp60 (*Ctr*) served as infection control. The immunoblots are representative of three independent experiments.

**Figure S7:** ***Mcl-1 is not relevant for the*** ***rapid Bax-diffusion away from mitochondria from* Ctr*-infected cells***

**A**. Isolated Mitochondria from either non-infected- or *Ctr*-infected HeLa cells deficient in Mcl-1 were re-suspended in MB-EGTA buffer and incubated for various times at 30°C. Reactions were pelleted by centrifugation and, the supernatants (S) or the pellets (P) were collected and subjected to immunoblotting. The fractions S and P were analyzed for the presence of Bax. Hsp60 served as a loading control for mitochondria.

**B**. FLIP measurements of GFP-BAX following apoptosis induction by 1 µM staurosporine in the absence of *Ctr*-infection. Lines represent relative GFP-BAX fluorescence on the mitochondria of individual targeted cells compared to the average of unbleached control cells (straight line). FLIP, Fluorescence Loss In Photobleaching.

**C**. FLIP measurements of GFP-BAX following *Ctr*-infection and apoptosis induction by 1 µM Staurosporine. Lines represent diminished relative GFP-BAX fluorescence on the mitochondria of targeted cells compared to the average of unbleached control cells (straight line). FLIP, Fluorescence Loss In Photobleaching.

**D**. FLIP of mitochondrial GFP-BAX in HCT116 following *Ctr*-infection and apoptosis induction by 1 µM Staurosporine. Cells were photobleached in the marked region (pink circle). Changes in fluorescence were detected in 4 areas (0 s pictures, left; ROIs M1 – M4) over the indicated time period. ROI measurement of a reference cell (black circle) served as control.

**E**. FLIP of mitochondrial GFP-BAX in HCT116 following apoptosis induction by 1 µM staurosporine in the absence of chlamydia infection. Cells were photobleached in the marked region (pink circle). Changes in fluorescence were detected in 4 areas (0 s pictures, left; ROIs M1 – M4) over the indicated time period. ROI measurement of a reference cell (black circle) served as control.

**Figure S8:** ***In* Ctr*-infected HeLa cells, Bak forms a complex with VDAC and is not found in lower molecular weight upon apoptotic stimulus***

**A**- **B**. Bax-deficient HeLa cells were *Ctr*-infected (MOI=5) for 24h. Mitochondria were isolated and incubated either without or with tBid to induce Bak conformational changes and cytochrome *c* release. Reactions were centrifuged at 10,000xg and supernatants were analyzed by SDS-PAGE and Western blotting to detect the release of cytochrome *c* (bottom left). For **A**, pellets were resuspended in buffer containing 0.2 mM Cacl_2_ and incubated with calpain (20 nM) for 30 min at room temperature. Bak cleavage was detected by Western blotting with anti-Bak-G23 antibody. Full-length Bak and one cleavage fragment are indicated with arrows. The immunoblots are representative of two independent experiments. For **B,** pellet fractions were lysed in 1% digitonin-containing buffer for Blue Native (BN)-PAGE, and all the lysates were run on a 6% - 16.5% BN-PAGE gradient gel and subjected to immunoblotting for Bak and VDAC. The immunoblots are representative of three independent experiments.

**C**. Isolated mitochondria from either non-infected or *Ctr*-infected HeLa wt cells were either incubated on ice or at 30°C for 45 min. Samples were centrifuged and supernatants (S_1_) were collected. The pellets were subjected to sodium carbonate extraction and pellets (P) and supernatants (S_2_) were collected. All the different fractions P, S_2_ and S_1_ were boiled in Laemmli-buffer. The samples were immunoblotted for cytochrome *c*, Hsp60 and Bak. VDAC as mitochondrial integral protein serves as a marker for mitochondrial membranes.

**Figure S9:** ***Bak does not re-distribute with pro-survival Bcl-2 family protein upon apoptosis stimulation of* Ctr*-infected cells***

**A**- **B**. Cells were *Ctr*-infected (MOI=5, 24 h) as indicated. For **A**, HeLa cells were left untreated or treated with 1 µM staurosporine for 5 h in the presence of the caspase inhibitor QVD-OPh (10 µM). For **B**, cells were kept untreated. Cells were harvested, lysed in 1% CHAPS, and lysates were subjected to immunoprecipitation using Bak (Ab-1) antibody (**A**) and Bak (7D10) antibody (**B**). Input (whole cell-lysates), IP-reactions and supernatants of the reaction (‘unbound’) were loaded separately and assessed by Western blotting for Bak, Mcl-1, Bcl-X_L_, Bcl-2, VDAC2 and the chlamydial protein Hsp60(*Ctr*). GAPDH and α-tubulin serve as loading control. Data are representative of three independent experiments. α-tub (α-tubulin). Note that the antibody used in B recognizes Bak regardless of its activation state while the antibody used in A only recognizes activated Bak.

**Figure S10:** **Ctr*-infection Inhibits Bak-mediated apoptosis in the absence of VDAC2***

**A**. Western blot showed the results of deletion-efficiency of either VDAC2 and Bax or VDAC2 and Bak in HeLa cells. Whole cell-lysates were analyzed for Bax, Bak and VDAC2. α-tubulin served as loading control. The immunoblot is representative of three independent experiments.

**B**. HeLa cells wt or HeLa cells deficient in Bax/VDAC2 or Bak/VDAC2 were *Ctr*-infected of left non-infected. After 24 h cells were treated with the combination of the inhibitors of Bcl-2/Bcl-XL (ABT-737, 1 µM) and Mcl-1 (s63845, 0.5 µM) for an additional 4 h. Apoptosis was detected by staining for active caspase-3 and analysis by flow cytometry. Data are means/SEM from three independent experiments. ****, p ˂ 0.0001, **, p ˂ 0.01, two-way Anova for multiple comparisons.

**C**. Apoptosis was measured by active caspase-3 staining. Cells of indicated genotype were either uninfected or *Ctr*-infected with MOI of 5 for 24h, and then treated with the combination of 1µM ABT+ 0.5µM S63845 for 6h. Results are expressed as means/SEM of three independent experiments. ***, p ˂ 0.001, ns (not significant), two-way Anova for multiple comparisons.

**Table legend**

**Table S1:** ***Characteristics of Bak antibodies used to characterize Bak conformational changes***
