# Supplementary figures and images for: Chlamydia trachomatis inhibits apoptosis in infected cells by targeting the pro-apoptotic proteins Bax and Bak
Source: Cell Death Differ. 2022 Apr 9;29(10):2046–59. doi: 10.1038/s41418-022-00995-0 (PMC9525694; doi:10.1038/s41418-022-00995-0)

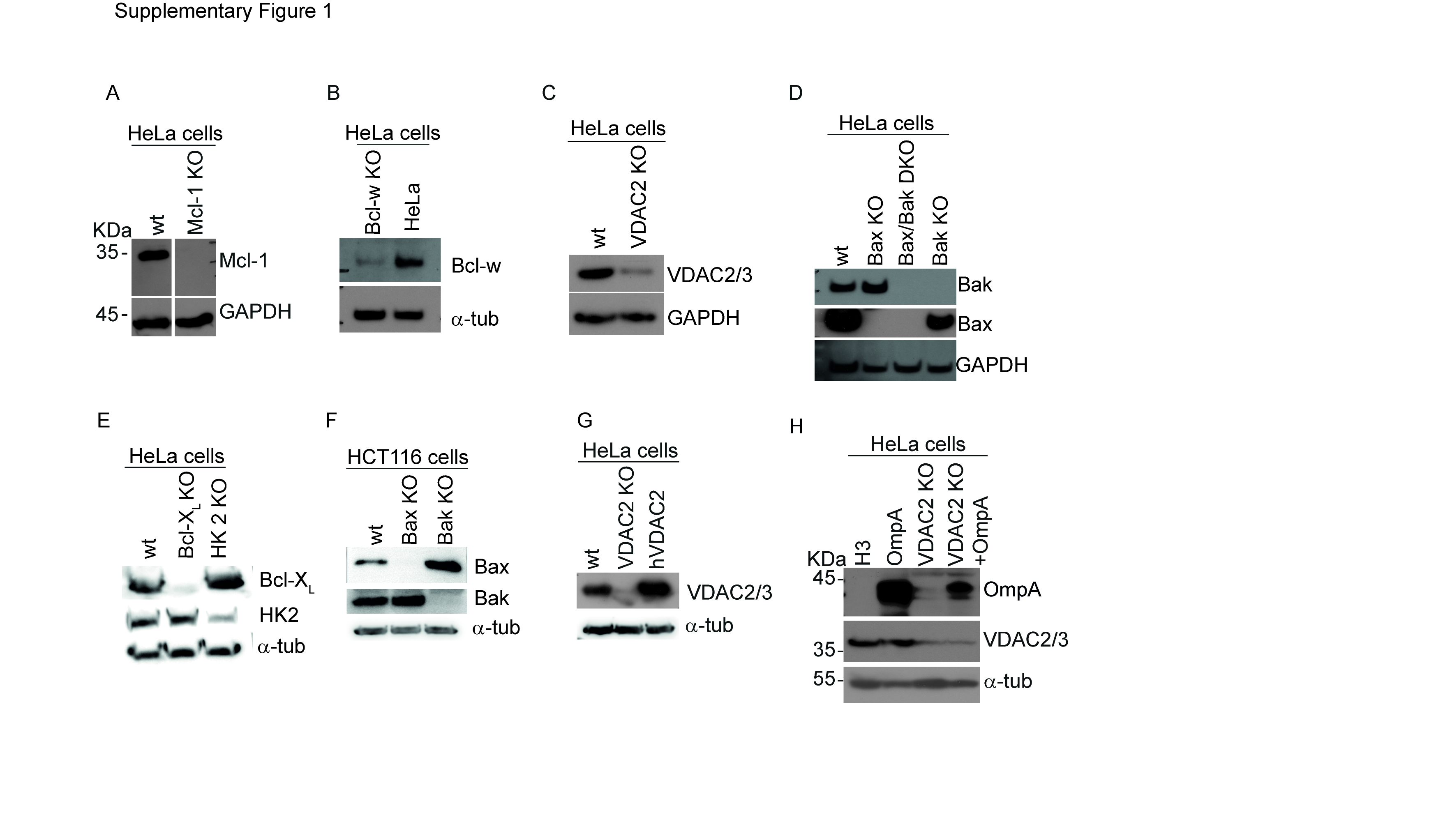

Supplement: Supplementary file 2 — Figure S1 [file 41418_2022_995_MOESM2_ESM.tif]

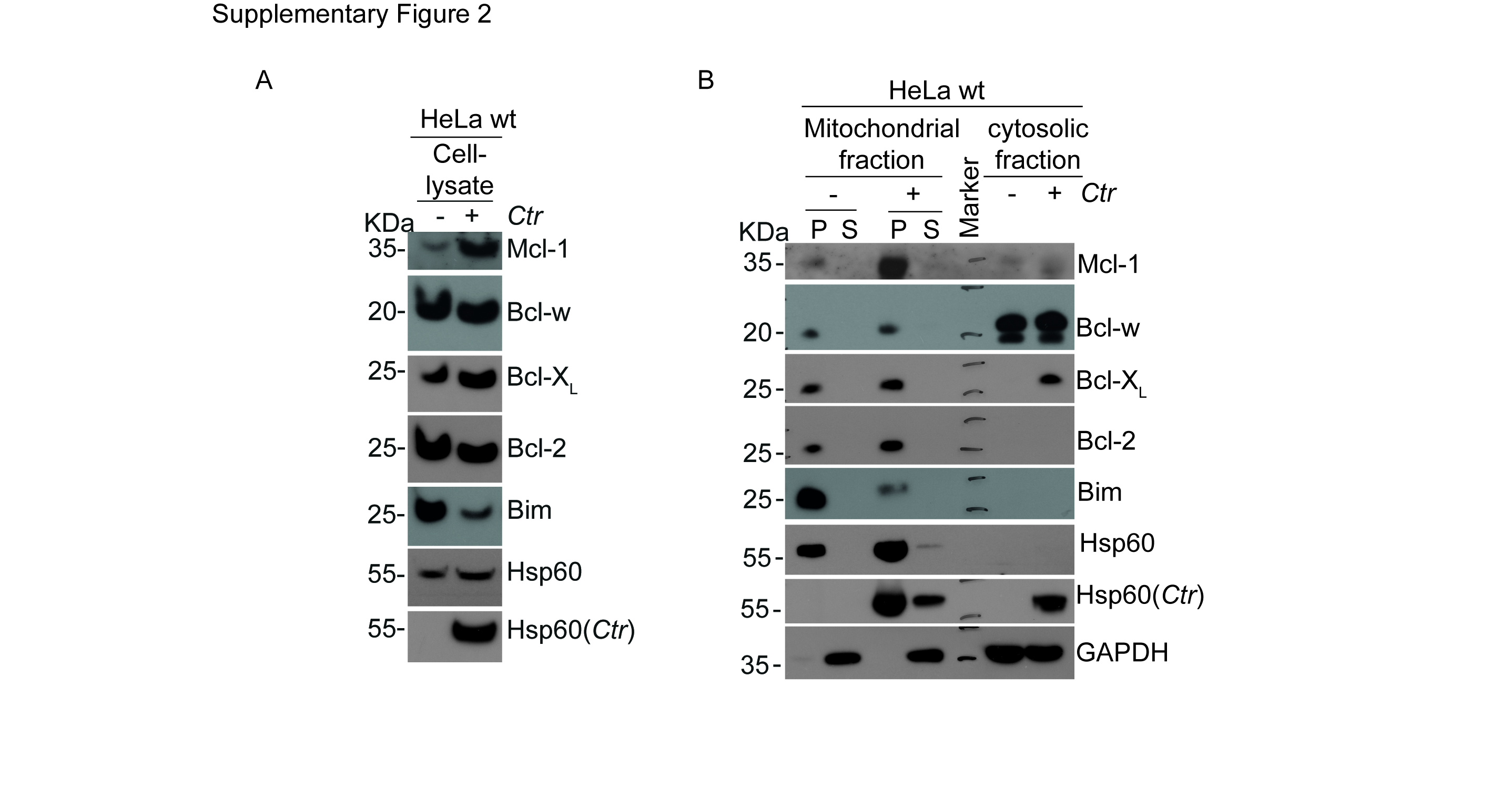

Supplement: Supplementary file 3 — Figure S2 [file 41418_2022_995_MOESM3_ESM.tif]

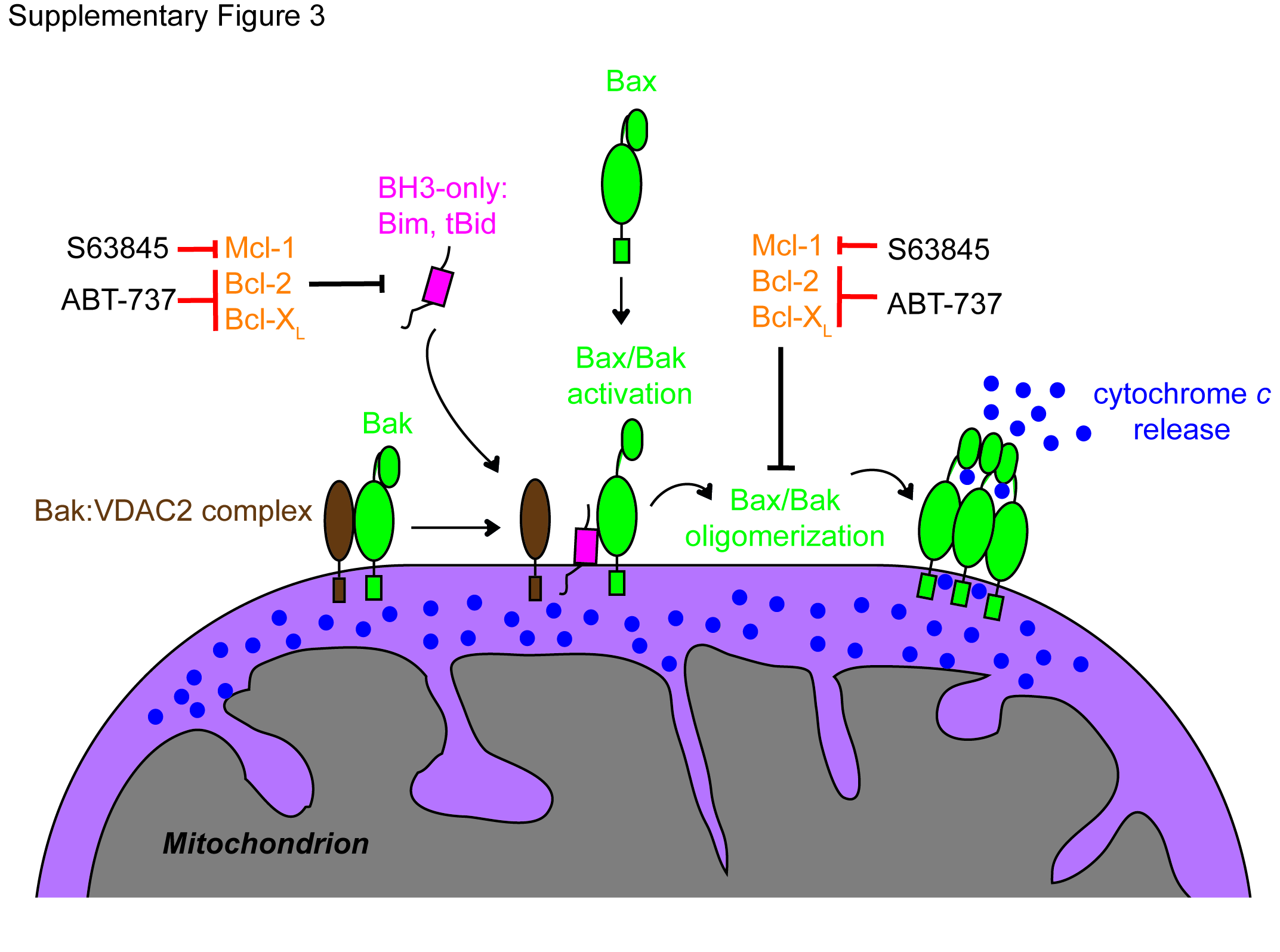

Supplement: Supplementary file 4 — Figure S3 [file 41418_2022_995_MOESM4_ESM.tif]

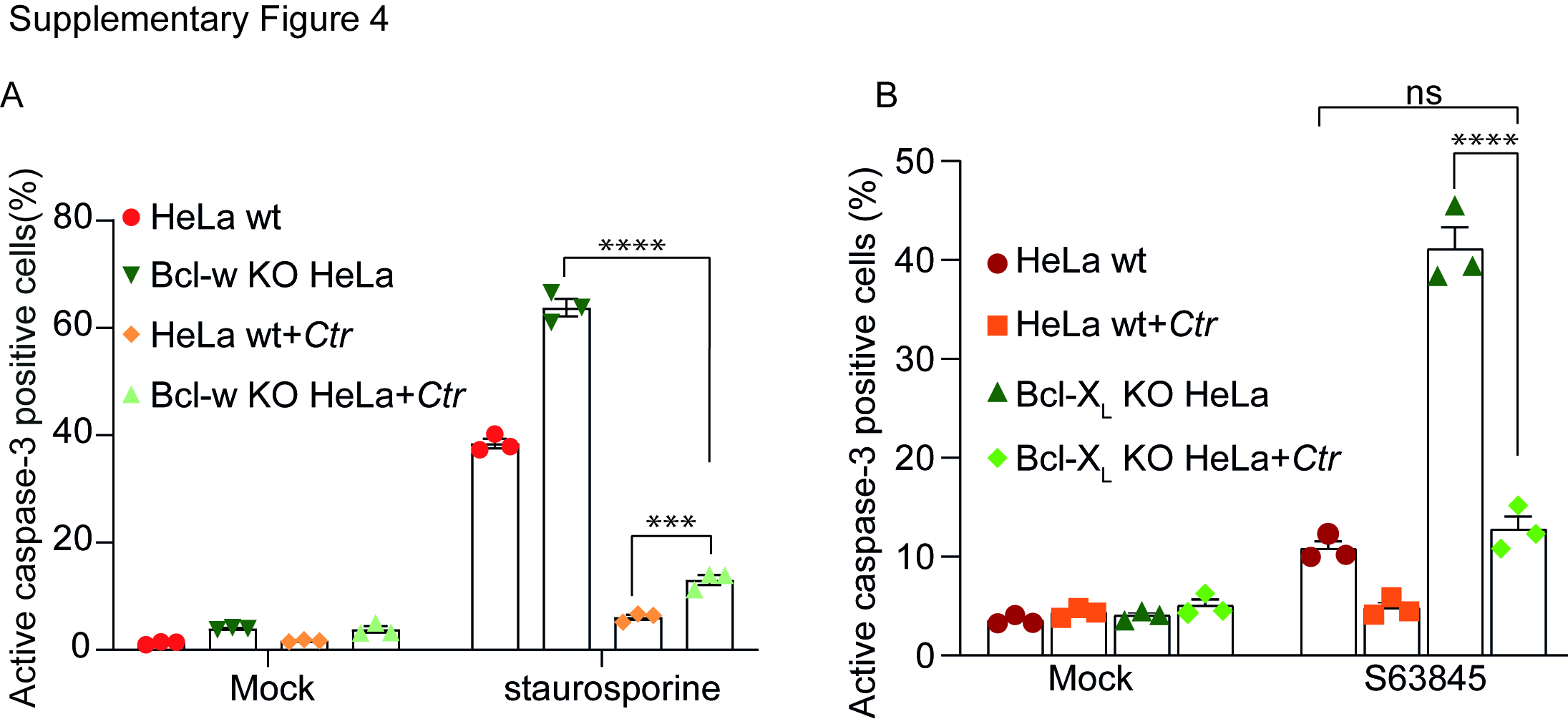

Supplement: Supplementary file 5 — Figure S4 [file 41418_2022_995_MOESM5_ESM.tif]

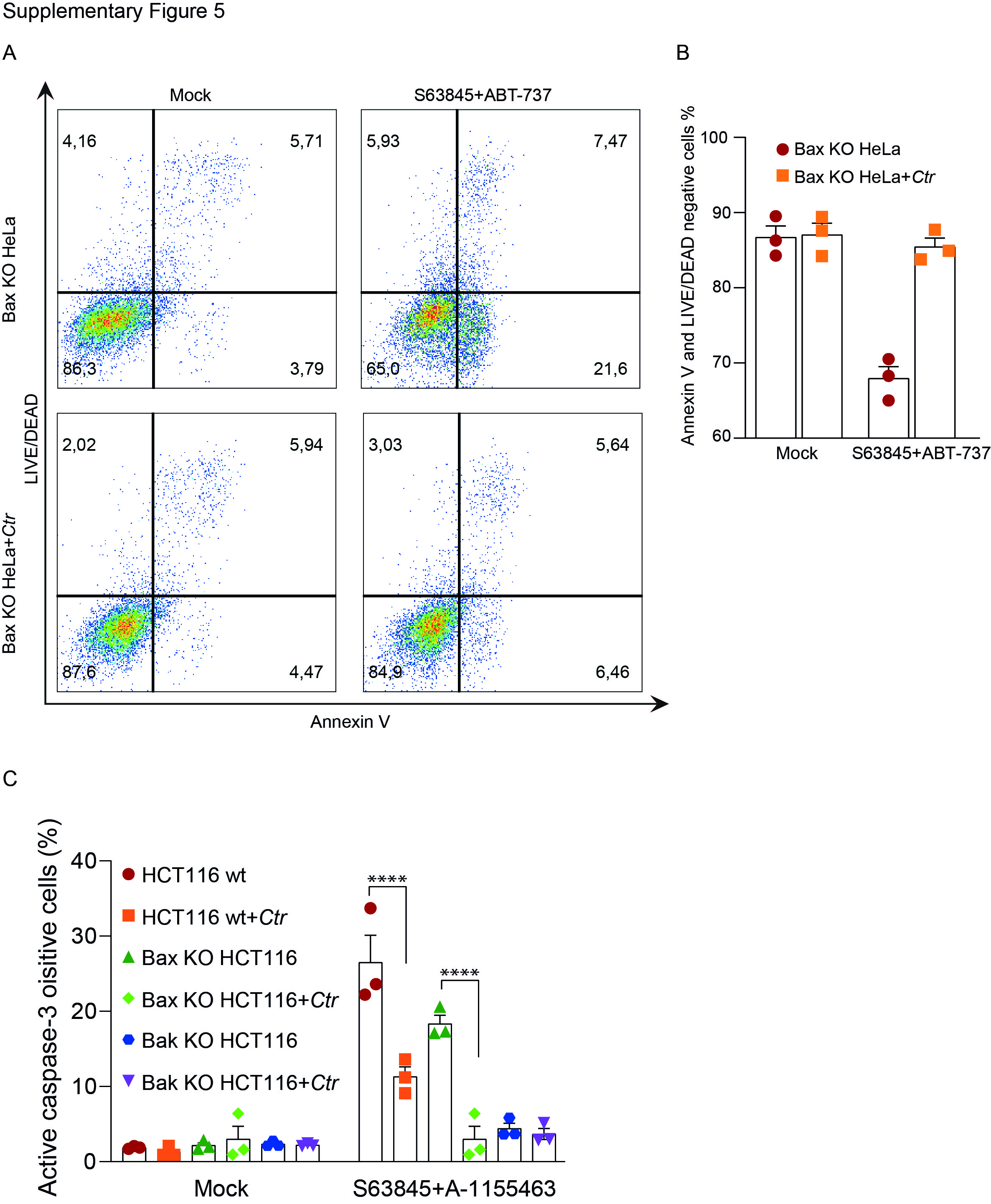

Supplement: Supplementary file 6 — Figure S5 [file 41418_2022_995_MOESM6_ESM.jpg]

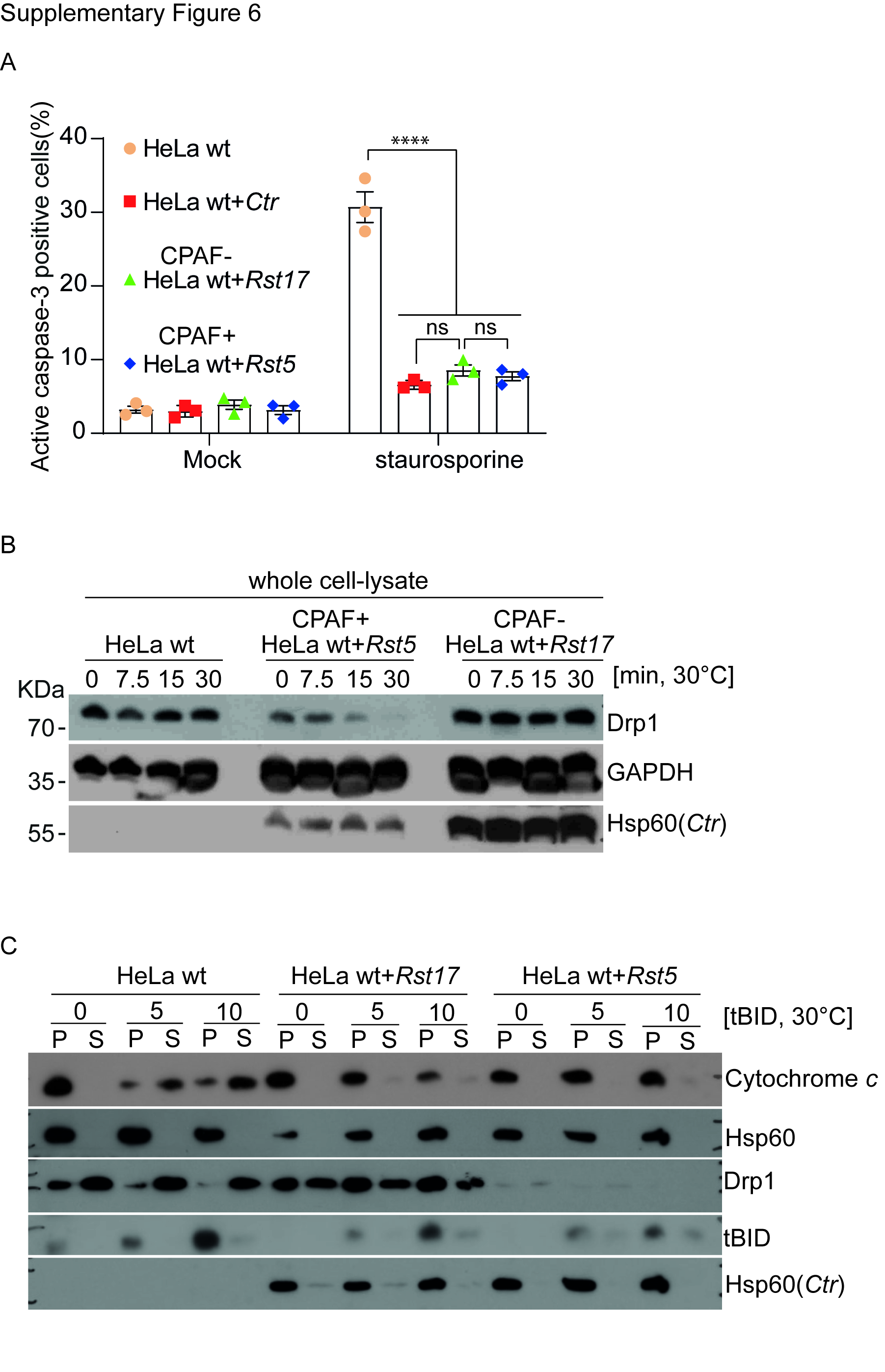

Supplement: Supplementary file 7 — Figure S6 [file 41418_2022_995_MOESM7_ESM.tif]

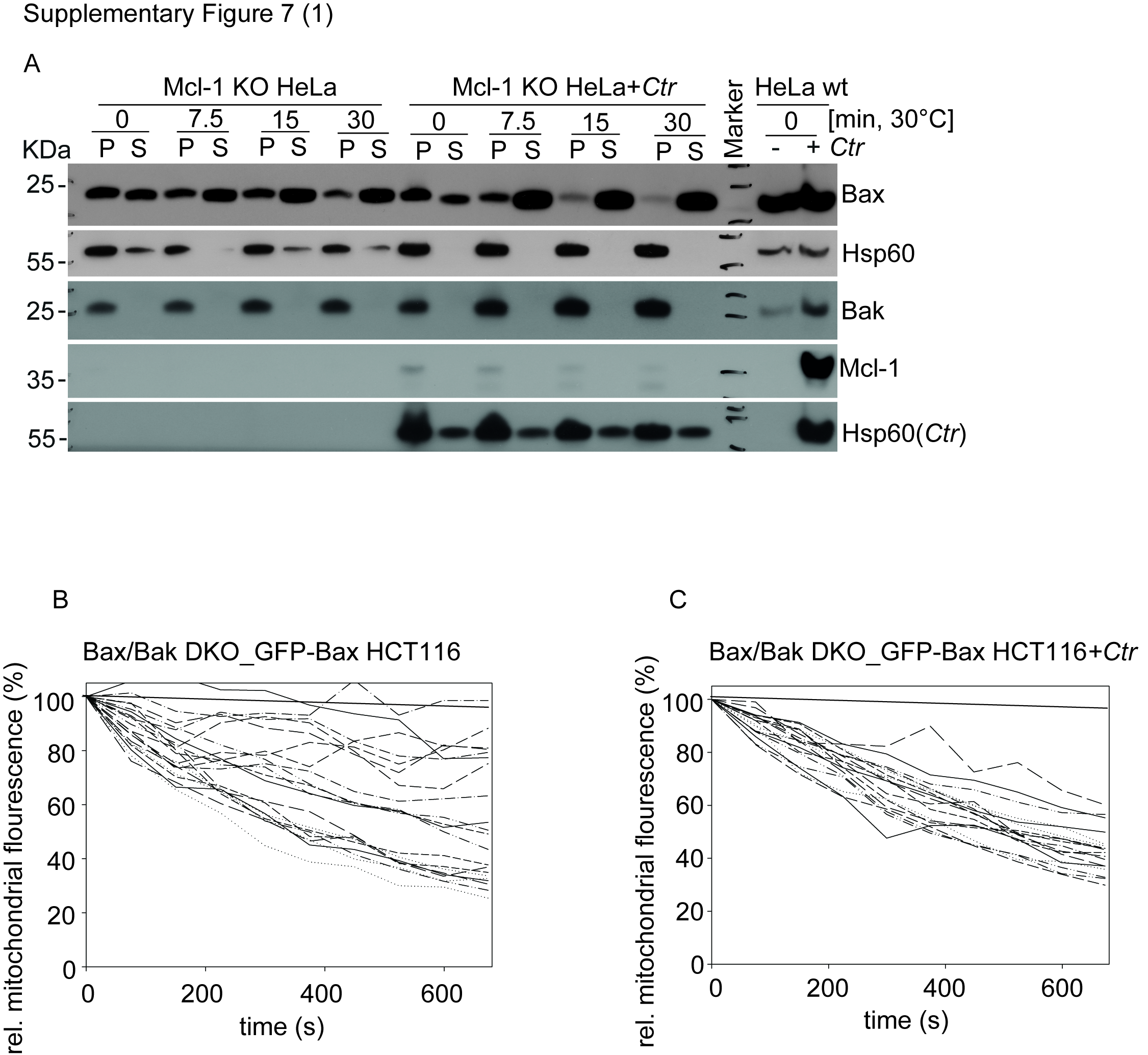

Supplement: Supplementary file 8 — Figure S7_1 [file 41418_2022_995_MOESM8_ESM.tif]

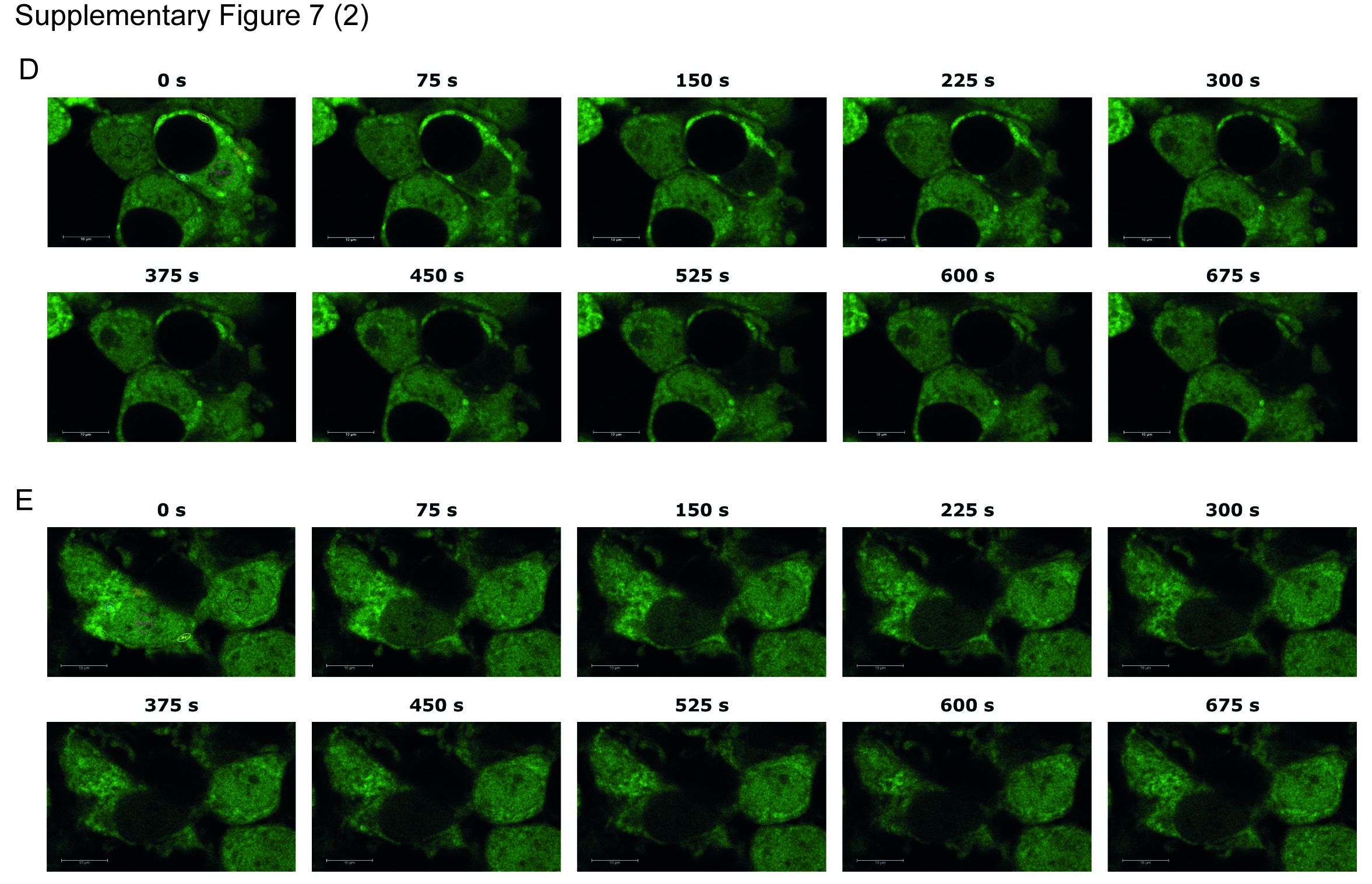

Supplement: Supplementary file 9 — Figure S7_2 [file 41418_2022_995_MOESM9_ESM.tif]

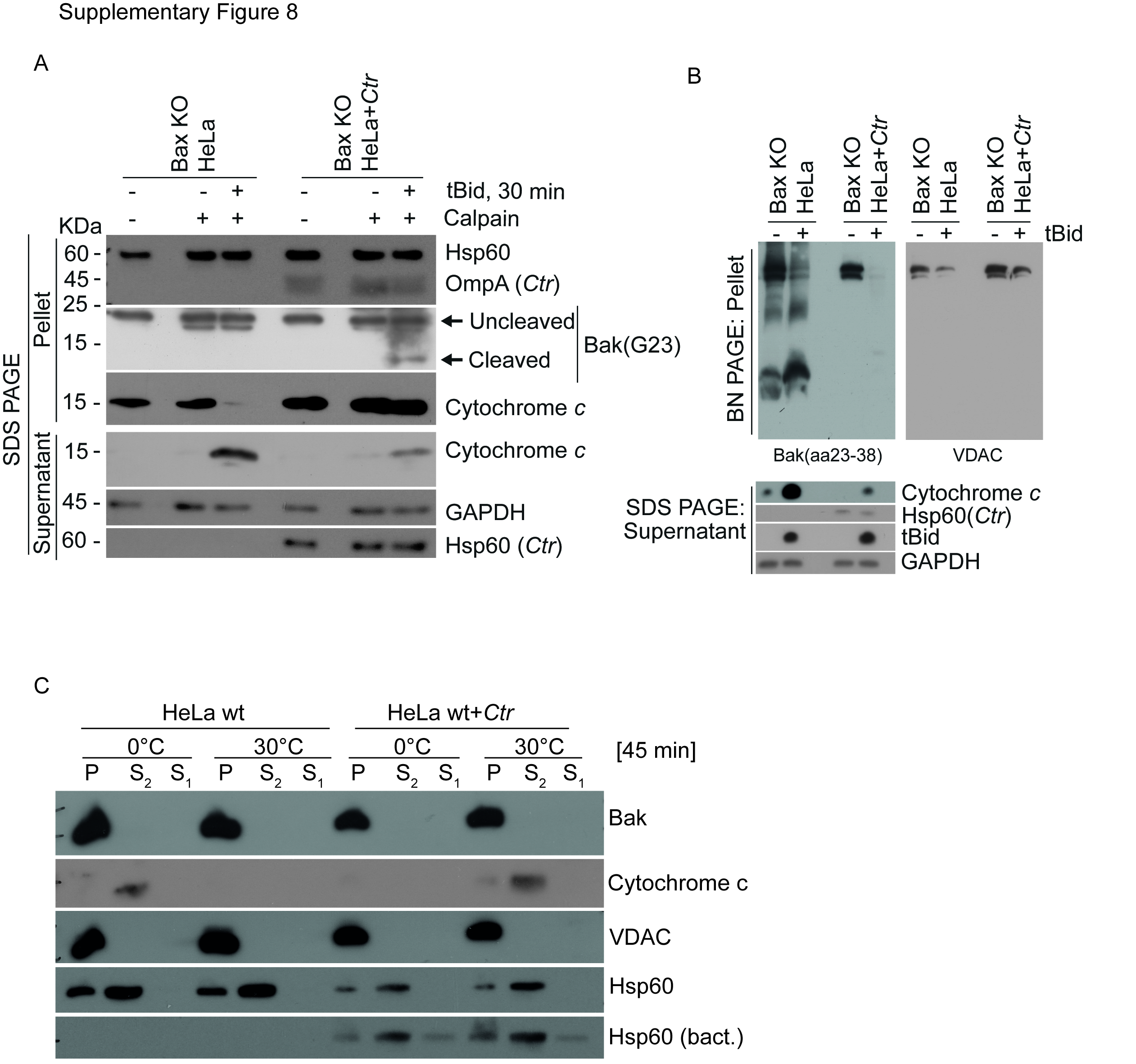

Supplement: Supplementary file 10 — Figure S8 [file 41418_2022_995_MOESM10_ESM.tif]

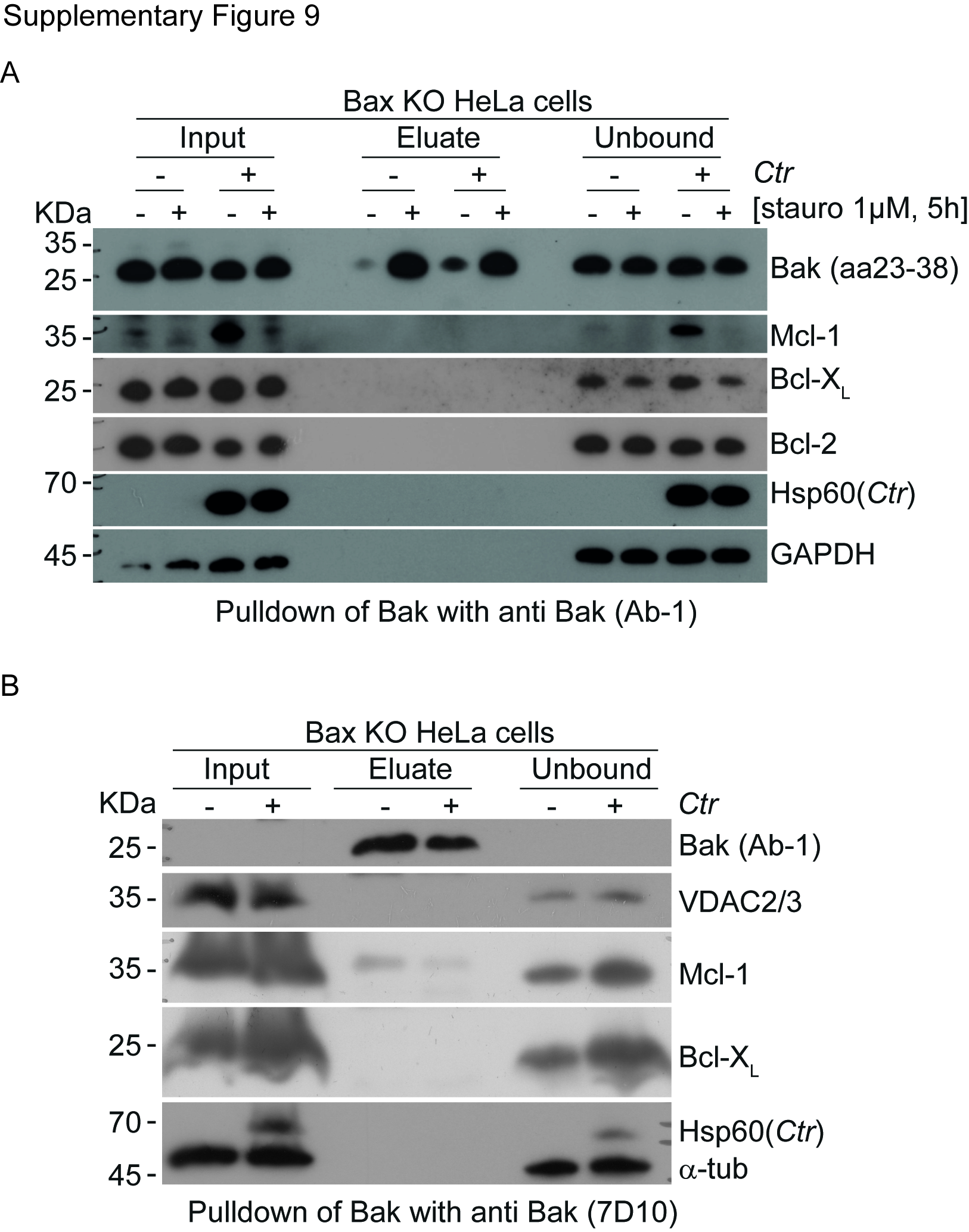

Supplement: Supplementary file 11 — Figure S9 [file 41418_2022_995_MOESM11_ESM.tif]

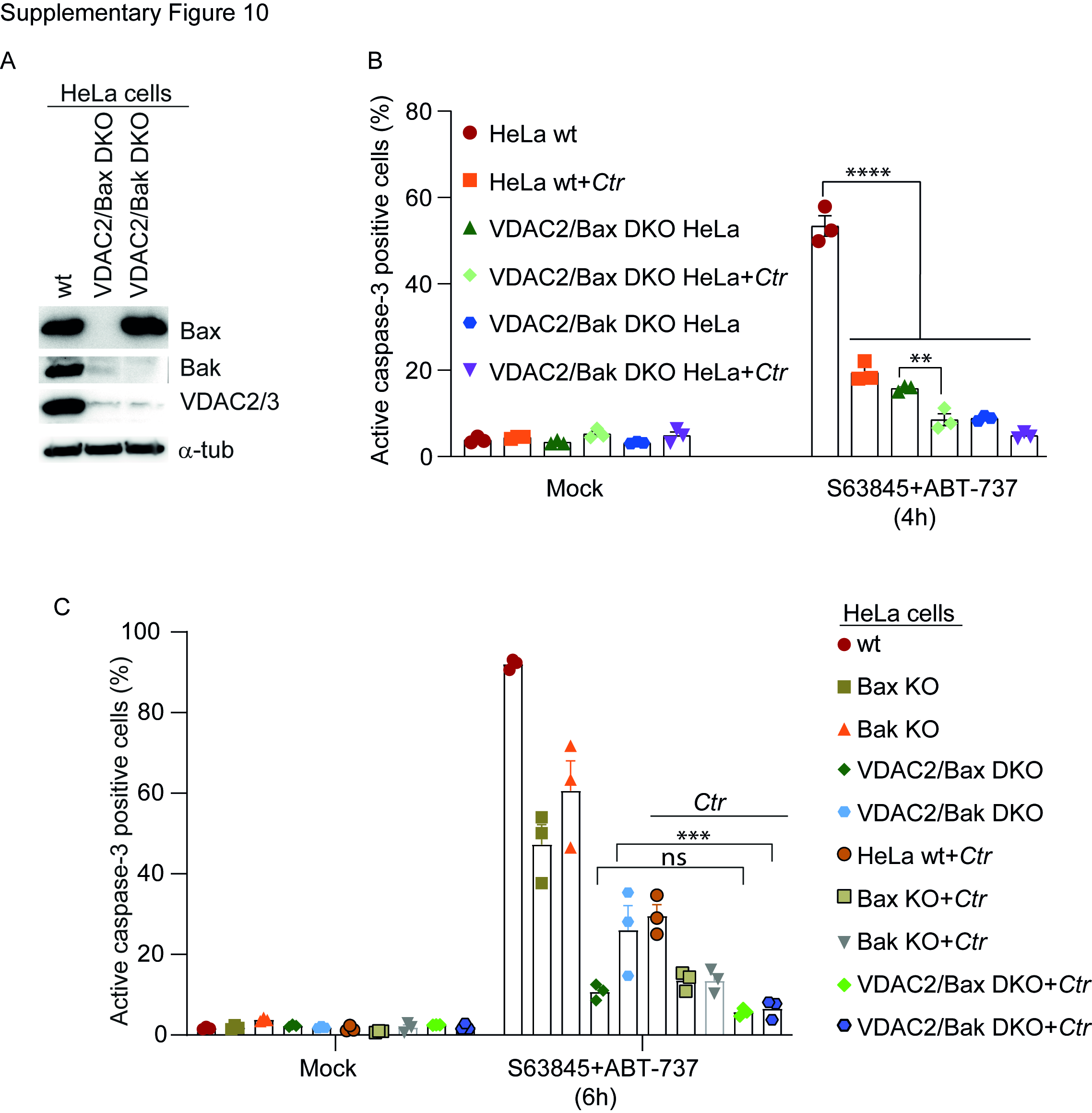

Supplement: Supplementary file 12 — Figure S10 [file 41418_2022_995_MOESM12_ESM.tif]

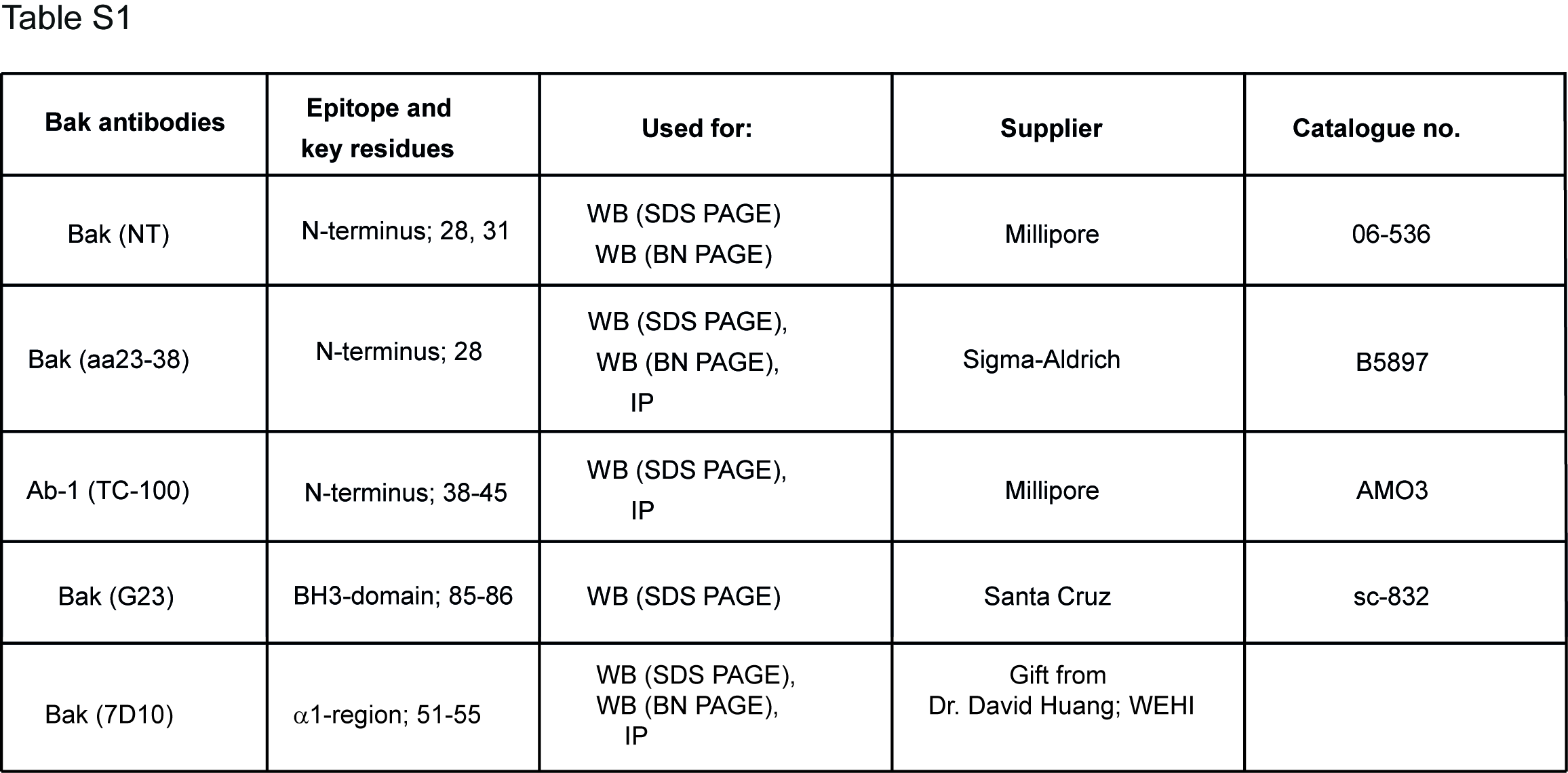

Supplement: Supplementary file 13 — Table S1 [file 41418_2022_995_MOESM13_ESM.tif]

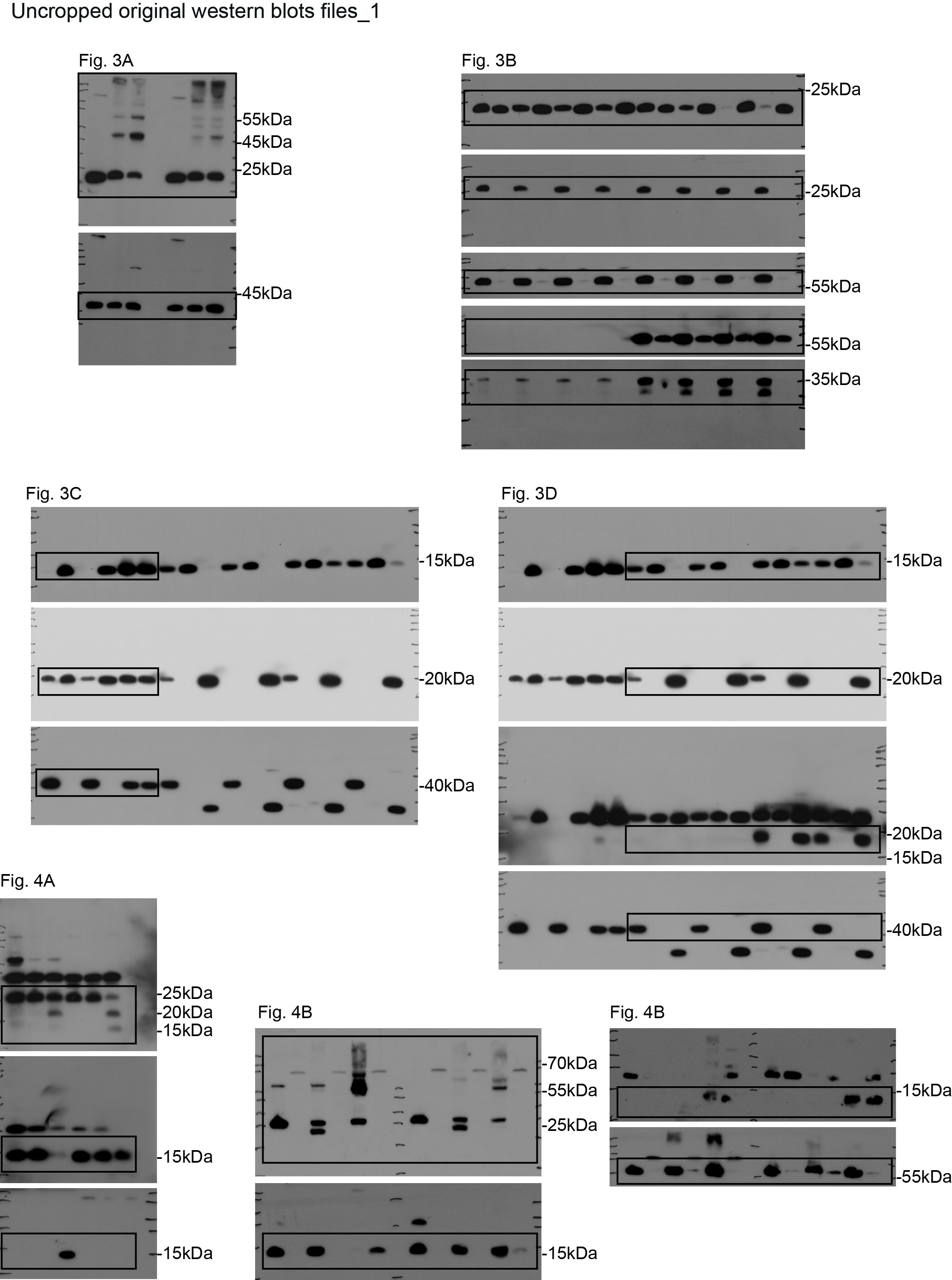

Supplement: Supplementary file 15 — Uncropped original western blots files [file 41418_2022_995_MOESM15_ESM.jpg]

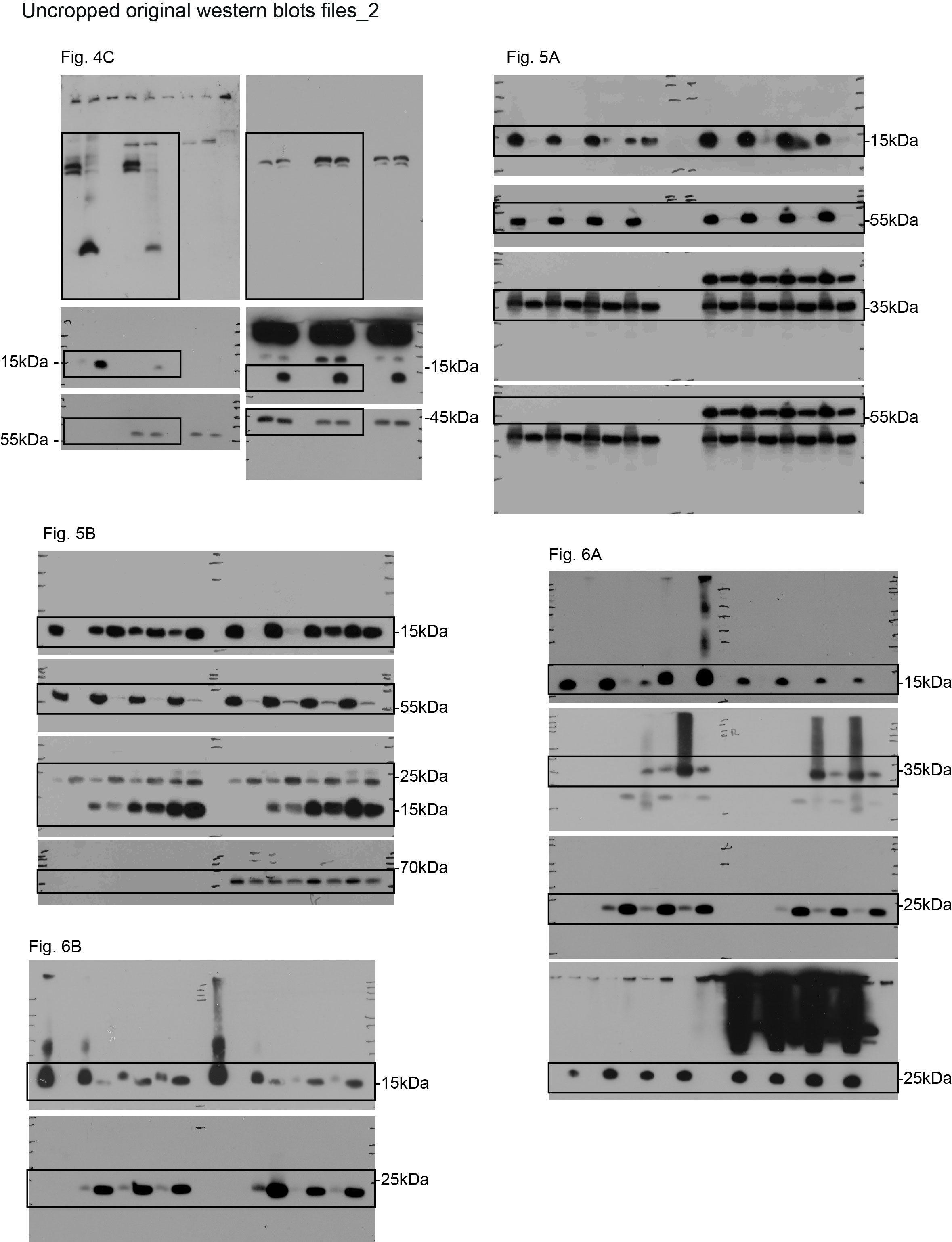

Supplement: Supplementary file 16 — Uncropped original western blots files [file 41418_2022_995_MOESM16_ESM.jpg]

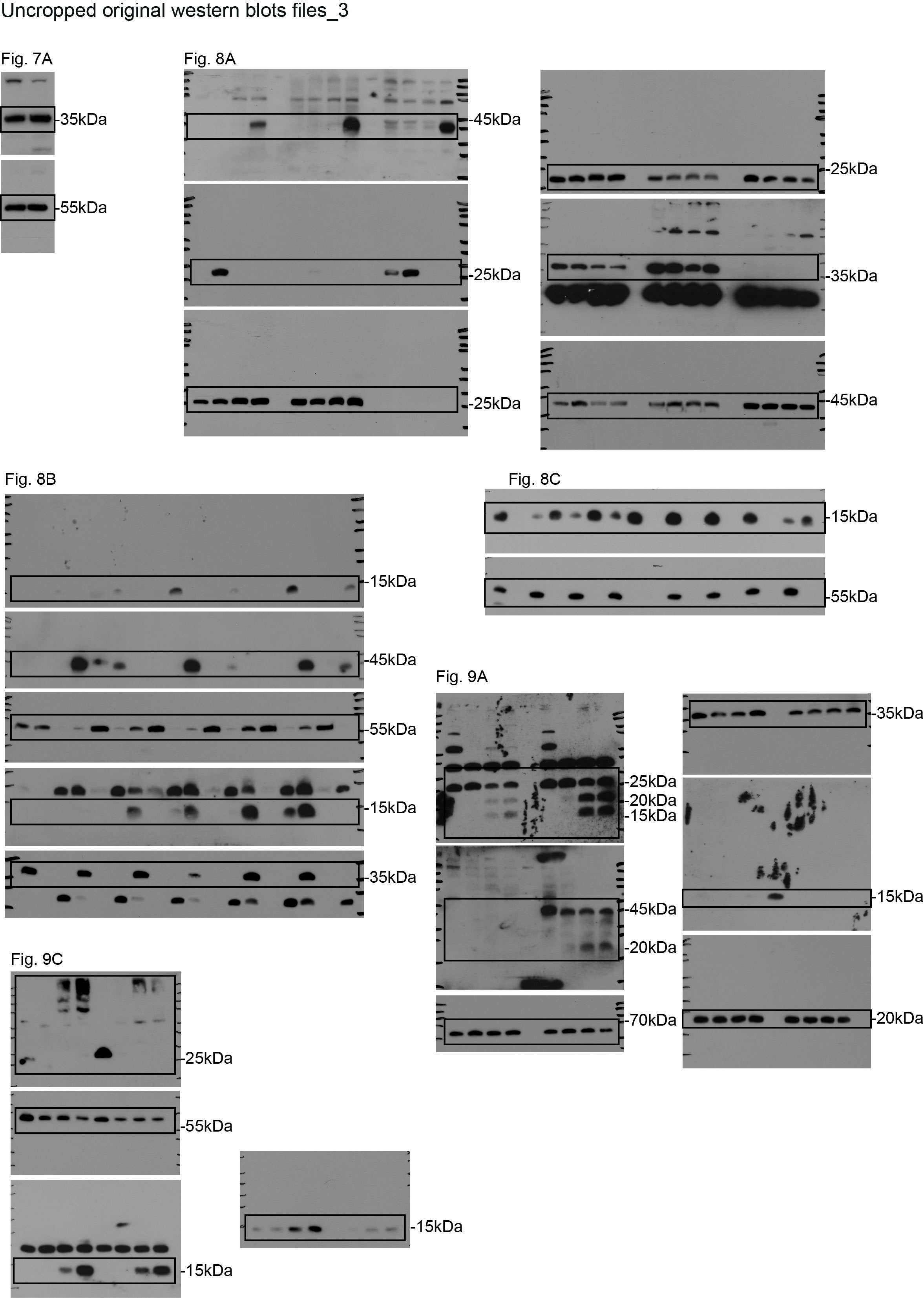

Supplement: Supplementary file 17 — Uncropped original western blots files [file 41418_2022_995_MOESM17_ESM.jpg]

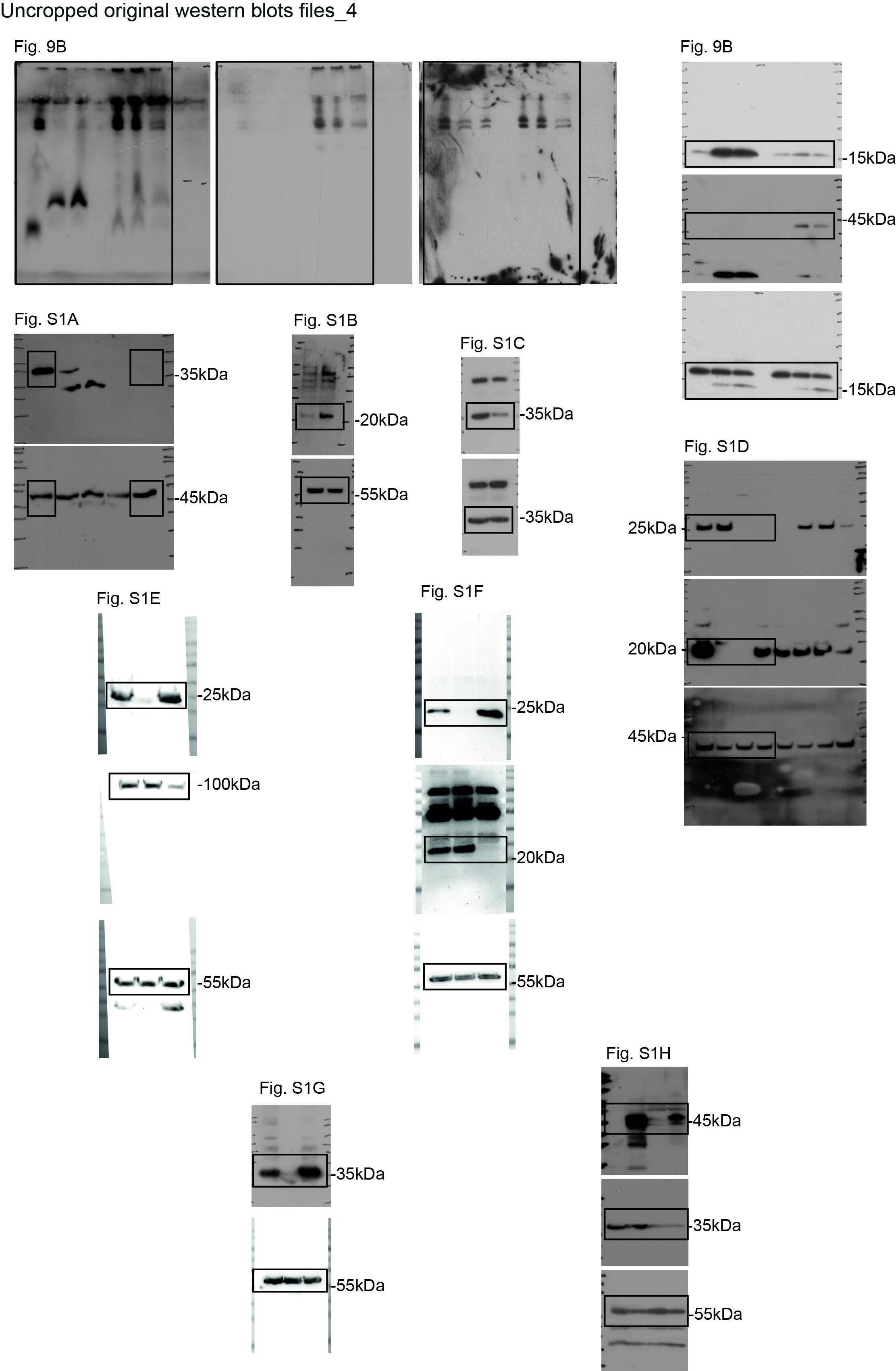

Supplement: Supplementary file 18 — Uncropped original western blots files [file 41418_2022_995_MOESM18_ESM.jpg]

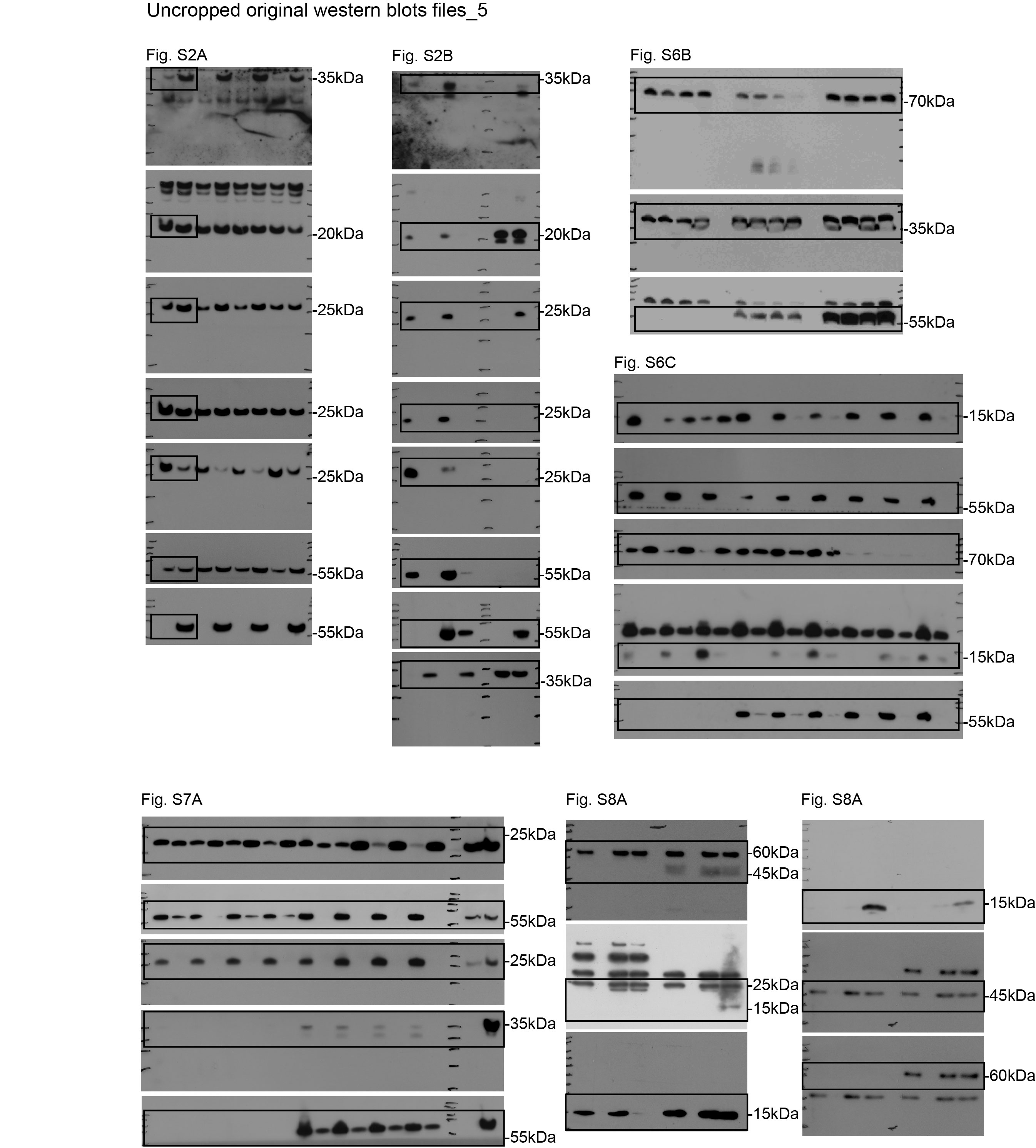

Supplement: Supplementary file 19 — Uncropped original western blots files [file 41418_2022_995_MOESM19_ESM.jpg]

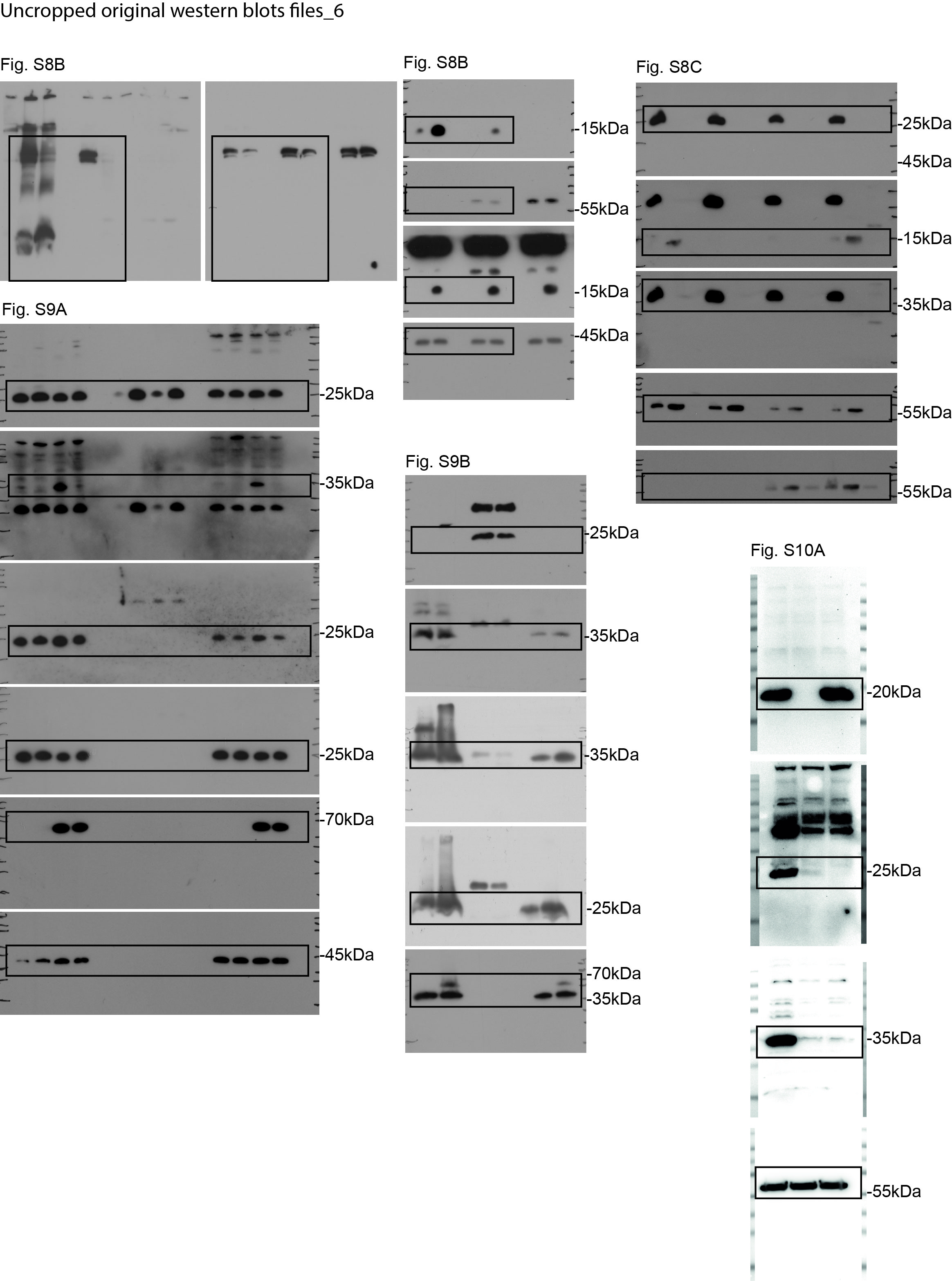

Supplement: Supplementary file 20 — Uncropped original western blots files [file 41418_2022_995_MOESM20_ESM.jpg]
